# Essentiality of the Maltase AmlE in Maltose Utilization and Its Transcriptional Regulation by the Repressor AmlR in the Acarbose-Producing Bacterium *Actinoplanes* sp. SE50/110

**DOI:** 10.3389/fmicb.2019.02448

**Published:** 2019-10-29

**Authors:** Lena Schaffert, Susanne Schneiker-Bekel, Saskia Dymek, Julian Droste, Marcus Persicke, Tobias Busche, David Brandt, Alfred Pühler, Jörn Kalinowski

**Affiliations:** ^1^Microbial Genomics and Biotechnology, Center for Biotechnology, Bielefeld University, Bielefeld, Germany; ^2^Senior Research Group in Genome Research of Industrial Microorganisms, Center for Biotechnology, Bielefeld University, Bielefeld, Germany

**Keywords:** PurR/LacI-type transcriptional regulator, maltase MalL, *amlE-amlR* gene arrangement, *acb* and *gac* gene cluster, maltose/maltodextrin metabolism, hydrolase assay, phosphorylase assay, secondary effects by c-di-GMP metabolism

## Abstract

*Actinoplanes* sp. SE50/110 is the wild type of industrial production strains of the fine-chemical acarbose (acarviosyl-maltose), which is used as α-glucosidase inhibitor in the treatment of type II diabetes. Although maltose is an important building block of acarbose, the maltose/maltodextrin metabolism has not been studied in *Actinoplanes* sp. SE50/110 yet. Bioinformatic analysis located a putative maltase gene *amlE* (*ACSP50_2474*, previously named *malL*; Wendler et al., 2015a), in an operon with an upstream PurR/LacI-type transcriptional regulator gene, named *amlR* (*ACSP50_2475*), and a gene downstream (*ACSP50_2473*) encoding a GGDEF-EAL-domain-containing protein putatively involved in c-di-GMP signaling. Targeted gene deletion mutants of *amlE* and *amlR* were constructed by use of the CRISPR/Cas9 technology. By growth experiments and functional assays of Δ*amlE*, we could show that AmlE is essential for the maltose utilization in *Actinoplanes* sp. SE50/110. Neither a gene encoding a maltose phosphorylase (MalP) nor MalP enzyme activity were detected in the wild type. By this, the maltose/maltodextrin system appears to be fundamentally different from other described prokaryotic systems. By sequence similarity analysis and functional assays from the species *Streptomyces lividans* TK23, *S. coelicolor* A3(2) and *S. glaucescens* GLA.O, first hints for a widespread lack of MalP and presence of AmlE in the class Actinobacteria were given. Transcription of the *aml* operon is significantly repressed in the wild type when growing on glucose and repression is absent in an Δ*amlR* deletion mutant. Although AmlR apparently is a local transcriptional regulator of the *aml* operon, the Δ*amlR* strain shows severe growth inhibitions on glucose and – concomitantly – differential transcription of several genes of various functional classes. We ascribe these effects to *ACSP50_2473*, which is localized downstream of *amlE* and presumably involved in the metabolism of the second messenger c-di-GMP. It can be assumed, that maltose does not only represent the most important carbon source of *Actinoplanes* sp. SE50/110, but that its metabolism is coupled to the nucleotide messenger system of c-di-GMP.

## Introduction

*Actinoplanes* sp. SE50/110 (ATCC 31044) is a natural derivative of SE50, which was isolated from a soil sample during a screening program by the Bayer AG in 1970 (Frommer et al., 1979; Parenti and Coronelli, 1979). It was found to be a natural producer of the α-glucosidase inhibitor acarbose, which is used in the treatment of diabetes mellitus (Wehmeier, 2004; Wehmeier and Piepersberg, 2004). After oral application of acarbose, human intestinal α-glucosidases are inhibited, which leads to a retarded release of monosaccharides. By this, the postprandial blood sugar levels in diabetes patients can be substantially decreased, which is beneficial in the context of cardiovascular morbidity (Rosak and Mertes, 2012).

Acarbose is a cyclitol-containing aminoglycoside. The biosynthesis genes are encoded by the *acb* gene cluster, which was identified in 1999 by Stratmann et al. and subsequently sequenced (GenBank: Y18523.4) (Stratmann et al., 1999; Thomas, 2001). Acarbose is composed of a pseudodisaccharide and an α-1,4-glycosidic-bound maltose (Wehmeier and Piepersberg, 2009).

In the context of saving resources from the extracellular space, the secondary metabolite acarbose has been assumed to have a double function for *Actinoplanes* sp. SE50/110 – especially when being in competing situations within its natural habitat soil: It was assumed to function as inhibitor of foreign glucosidases on the one hand and as “carbophore” on the other hand (Wehmeier and Piepersberg, 2004; Brunkhorst and Schneider, 2005). “Carbophore” describes the model idea of acarbose as transport vehicle, on which sugars can be transferred. By this, sugar molecules of the environment are tagged for reimport and detracted from other bacteria (Wehmeier and Piepersberg, 2004, 2009). After reimport, acarviosyl-metabolites are probably phosphorylated and unloaded leading to the recirculation of acarbose (Wehmeier and Piepersberg, 2004; Wendler et al., 2013). By its double function, acarbose assures growth advantages of the slowly growing *Actinoplanes* sp. SE50/110 in a diverse microbial community.

The disaccharide maltose is substantial for the formation of acarbose, because it serves as central precursor of the acarbose biosynthesis on the one hand and as carbon source on the other hand. Therefore, the uptake from the extracellular space, the intracellular utilization and the regulation are of utmost interest for the research on *Actinoplanes* sp. SE50/110. In contrast to the extracellular sugar metabolism, only little is known about the intracellular metabolism (Ortseifen, 2016) and its regulation (Wolf, 2017) in *Actinoplanes* sp. SE50/110.

According to the current models from the well-studied microorganisms Gammaproteobacterium *Escherichia coli* and Actinobacterium *Corynebacterium glutamicum*, the maltose/maltodextrin system is designated by the activity of the two key enzymes: The amylomaltase MalQ and the phosphohydrolase MalP. In *E. coli*, MalQ recognizes small maltodextrins, removes glucose from the reducing end and transfers the maltodextrinyl-residue to the non-reducing end of other maltodextrins (Boos and Shuman, 1998; Park et al., 2011). As consequence, larger linear maltodextrins are built up by the MalQ activity, whereas glucose is released. Maltotriose is the smallest substrate of MalQ (Boos and Shuman, 1998; Park et al., 2011). Maltose is no primary substrate, but can serve as acceptor of transfer reactions (Boos and Shuman, 1998). MalP is assumed to degrade linear glucans formed by MalQ by sequential phosphorolysis. By this, glucose-1P is formed, which is directed to the glycolysis for growth and energy production (Boos and Shuman, 1998; Park et al., 2011). MalP does not attack maltodextrins of a size below maltotetraose, probably since dextrins of a minimum size are required for MalQ (Boos and Shuman, 1998; Park et al., 2011). For *E. coli*, the interplay between MalQ and MalP allows for an efficient utilization of maltodextrins of different size in the cell (Boos and Shuman, 1998; Park et al., 2011). Similar findings have been reported for the actinobacterium *C. glutamicum* (Seibold et al., 2009).

Further glucosidases, namely MalZ and MalS, are assumed to degrade linear glucans in *E. coli*. Both enzymes release glucose from the reducing end with maltotriose as smallest substrate (Tapio et al., 1991; Boos and Shuman, 1998). In *E. coli*, MalZ is located in the cytoplasm whereas MalS is located in the periplasm (Boos and Shuman, 1998). Here, MalS preferentially cleaves maltohexaose from the non-reducing end of higher maltodextrins to enable transport from the periplasm to the cytoplasm by maltose/maltodextrin transport systems (Boos and Shuman, 1998). By this, MalS is dedicated to Gram-negative bacteria (Boos and Shuman, 1998). Whereas the lack of MalP has dramatic physiological effects, MalZ and MalS are not essential for the maltose or maltodextrin utilization in *E. coli* (Park et al., 2011). In the Gram-positive bacterium *C. glutamicum*, a MalZ-homolog could not be identified yet (Seibold et al., 2009). Consequently – in both model organisms – MalP and MalQ have a leading role in the maltose assimilation, whereas MalZ or MalS seem to be negligible.

The maltose/maltodextrin system of the Gram-positive Firmicute bacterium *Bacillus subtilis* discriminates substantially from the models of *E. coli* and *C. glutamicum* as it lacks homologs of GlgX, MalQ, MalP, and MalZ (Shim et al., 2009). Their role in maltodextrin-assimilation is functionally replaced by the activity of a maltogenic amylase (MAase, YvdF), a pullulanase (AmyX), a maltose phosphorylase (YvdK), and a maltase, called MalL (Schönert et al., 1998, 2006; Shim et al., 2009).

In *Actinoplanes* sp. SE50/110 a maltase gene is annotated as well, which was previously named MalL (Wendler et al., 2015a). In this paper, we rename MalL into AmlE (*Actinoplanes* maltase Enzyme). AmlE attracts our attention, as it is highly expressed when growing on maltose (Wendler et al., 2015a). As no functional homolog of AmlE has been described in the maltose/maltodextrin system of the well-studied model organisms *E. coli* (Boos and Shuman, 1998; Park et al., 2011) and *C. glutamicum* (Seibold, 2008; Seibold et al., 2009), it was suggested, that AmlE is responsible for the breakdown of high molecular sugars into glucose by a hydrolytic activity – comparable to the hydrolase MalZ from *E. coli* (Ortseifen, 2016).

In the context of gene regulation, the maltose/maltodextrin system has been described as sugar-dependent– mostly maltose-induced or glucose-repressed-in different model organisms. In *E. coli*, the expression of *mal* genes is strictly maltose/maltotriose-induced and glucose-repressed. This is mediated by the master activator MalT, which is itself catabolite-repressed by cAMP/CAP as well as by the activity of the repressor Mlc (Boos and Shuman, 1998). In the Actinomycetes *Streptomyces lividans* (Schlösser et al., 2001) and *Streptomyces coelicolor* (van Wezel et al., 1997a, b), a MalR-homolog regulates the maltose-induced and glucose-repressed transcription of the transporter regulon *malEFG*. In *S. lividans*, MalR has even been described as pleiotropic regulator of various genes of sugar metabolism (Nguyen et al., 1997; Nguyen, 1999). In *B. subtilis*, the expression of the maltase MalL has been described as maltose-induced and glucose-/fructose-repressed (Schönert et al., 1998) together with other enzymes of the maltose/maltodextrin metabolism (Schönert et al., 1998, 2006). Here, the LacI/GalR-type regulator YvdE/MalR has been proposed as transcriptional activator (Schönert, 2004; Schönert et al., 2006).

In *Actinoplanes* sp. SE50/110, research about the regulation of sugar-metabolism is still at early phase. Here, over 900 genes are putatively involved in the transcriptional regulation, of which 697 are annotated as transcriptional regulators according to the annotation of Wolf et al. (2017b) (GenBank: LT827010.1). A sugar-dependent expression of *amlE* and the putative ABC-type transporter *aglEFG* were shown by comparative proteome analyses of *Actinoplanes* sp. SE50/110 grown on glucose and maltose (Wendler et al., 2015a), but the regulatory mechanisms remain unclear. Recent studies of the regulators MalT (*ACSP50_3915*) and a MalT-like regulator (*ACSP50_3917*) have pointed out, that none is responsible for the regulation of the maltose/maltodextrin metabolism genes in SE50/110 (personal communication Dr. Timo Wolf & Julian Droste). Likewise, it has been shown, that a MalR homolog is the transcriptional repressor of *acbDE* and *acbZ*, but not of the *malEFG*-operon in *Actinoplanes* sp. SE50/110 (Wolf et al., 2017a). The regulator has been assumed to have evolutionary changed the regulon from *malEFG* to the *acb* gene cluster and was renamed into acarbose regulator C (AcrC) (Wolf et al., 2017a). Further putative transcriptional regulators of the sugar metabolism have not been analyzed in *Actinoplanes* sp. SE50/110 yet.

Since the intracellular metabolism of maltose and its regulation is previously unexplored in *Actinoplanes* sp. SE50/110, we wanted to evaluate the function of the *Actinoplanes* maltase Enzyme AmlE (previously MalL) and the adjacent localized regulator, named AmlR (*Actinoplanes* maltase Regulator).

## Materials and Methods

### Software, Tools, and Databases Used for Bioinformatic Studies

Basic local alignments were performed with the Blast-interface^1^ (Altschul et al., 1990, 1997, 2005) of the National Center for Biotechnological Information (NCBI). A functional domain search of proteins was performed with the multiple sequence alignment tool CCD (Conserved Domain Database)^2^ (Marchler-Bauer and Bryant, 2004; Marchler-Bauer et al., 2010, 2015, 2017). TMHMM Server v. 2.0 was used for prediction of transmembrane helices in proteins^3^ (Krogh et al., 2001). Tree building and visualization was performed by help of the tool Blast tree view 1.17.5 of the NCBI and the software Dendroscope 3^4^ (Huson and Scornavacca, 2012).

EDGAR 2.0 was used for comparative genome studies of the *aml* gene cluster^5^ (Blom et al., 2016). The tool MEME was used for discovering of novel, ungapped motifs in a sequence^6^ (Bailey and Elkan, 1994; Bailey et al., 2009) and the tool FIMO for genome-wide scanning of the motif^7^ (Grant et al., 2011).

### Strains

All strains used in this paper are listed in Table 1.

**TABLE 1:** List of strains.

**Strain**	**Strain collection**	**NCBI reference sequence**	**References**
*Actinoplanes* sp. SE50/110	ATCC^®^31044	NZ_LT827010.1	Frommer et al., 1979; Parenti and Coronelli, 1979; Wolf et al., 2017b
*Escherichia coli* DH5αMCR	Mcr-deficient derivative of *E. coli* DH1	NC_017638.1	Grant et al., 1990
*Escherichia coli* ET12567/pUZ8002	–	–	Kieser et al., 2000
*Streptomyces lividans* TK23	Plasmid-free derivative of *S. lividans* 66	NZ_CP009124.1 (TK24 as representative genome)	Kieser et al., 2000
*Streptomyces coelicolor* A3(2) M145	ATCC^®^BAA-471D-5, plasmid-free derivative of *Streptomyces coelicolor* A3(2) ATCC^®^BAA-471	NC_003888.3	Dyson and Schrempf, 1987; Bentley et al., 2002
*Streptomyces glaucescens* GLA.O	DSM40922	NZ_CP009438.1	Ortseifen et al., 2015

### Media and Cultivation Conditions

#### Preparation of Glycerol Stocks and Spore Solutions of *Actinoplanes* sp. SE50/110

For the preparation of glycerol stocks, *Actinoplanes* sp. SE50/110 (ATCC 31044) was grown in the complex medium NBS (11 g⋅L^–1^ glucose × 1H_2_O, 4 g⋅L^–1^ peptone, 4 g⋅L^–1^ yeast extract, 1 g⋅L^–1^ MgSO_4_⋅7H_2_O, 2 g⋅L^–1^ KH_2_PO_4_, 4 g⋅L^–1^ K_2_HPO_4_) and mixed 2:3 with sterile 86% (v/v) glycerol. Glycerol stocks were stored at −80°C. For spore formation, 200–300 μL of a glycerol stock was grown on agar plates of soy flour medium (SFM-agar) [20 g⋅L^–1^ soy flour (SOBO^®^ Naturkost (Cologne, Germany)], 20 g⋅L^–1^ D-mannitol, 20 g⋅L^–1^ Bacto^TM^ agar (Becton-Dickinson, Heidelberg, Germany and 167 μL 10 N NaOH in tap water). Spores could be harvested after 5-7 days of incubation at 28°C by washing them off in 3 mL distilled water with a cotton swab.

#### Preparation of Minimal Medium

Strains of *Actinoplanes* sp. SE50/110 (ATCC 31044) were grown in maltose minimal medium (72.06 g⋅L^–1^ maltose⋅1H_2_O, 5 g⋅L^–1^ (NH_4_)_2_SO_4_, 0.184 g⋅L^–1^ FeCl_2_⋅4H_2_O, 5.7 g⋅L^–1^ Na_3_C_6_H_5_O_7_⋅2H_2_O, 1 g⋅L^–1^ MgCl_2_⋅6H_2_O, 2 g⋅L^–1^ CaCl_2_⋅2H_2_O, trace elements (final concentration: 1 μM CuCl_2_, 50 μM ZnCl_2_, 7.5 μM MnCl_2_) and phosphate buffer consisting of 5 g⋅L^–1^ each K_2_HPO_4_ and KH_2_PO_4_ in aqua distilled). For media preparation and filter sterilization the protocols of Wendler et al. were followed (Wendler et al., 2013, 2015b).

For substitution of the carbon source maltose, 79.2 g⋅L^–1^ glucose⋅1H_2_O or 72.0 g⋅L^–1^ C-Pur (Cerestar 01908, Cerestar GmbH, Krefeld, Germany) were used instead of maltose-monohydrate. Mixtures of maltose and glucose were prepared in the ratio of 90:10 and 50:50. The 90:10 mixture contains 64.85 g⋅L^–1^ maltose⋅1H_2_O and 7.9 g⋅L^–1^ glucose⋅1H_2_O. The 50:50 mixture contains 36.03 g⋅L^–1^ maltose⋅1H_2_O and 39.6 g⋅L^–1^ glucose⋅1H_2_O. For starch medium, a 4% (w/v) opalescent solution of “starch soluble” from Acros Organics (part of Thermo Fisher Scientific, Geel, Belgium) was generated. For this, sterile water was preheated to 90°C in a water bath and the weighed portion added with stirring. Afterward, the residual media components were added.

#### Shake Flask Cultivation

Cultivation was performed in 250 mL Corning^®^ Erlenmeyer baffled cell culture flasks at 28°C and 140 rpm for 7 days. For inoculation, a spore solution was prepared, of which 1 mL was used for inoculation of a 50 mL culture. Cell dry weights were determined by harvesting of 2 × 1 mL of the cell suspension in weighed reaction tubes (14,000 g, 2 min), washing them in deionized water and drying them for 1 day at 60–70°C, like described by Wolf et al. (2017a). The supernatant was stored at −20°C for later analyses.

### Acarbose Quantification

Supernatants of maltose-grown cultures of *Actinoplanes* ssp. were mixed 1:5 with methanol by vortexing and centrifuged to remove the precipitate (20,000 g, 2 min). The sample was transferred to HPLC vials and analyzed in the HPLC system 1100 series of Agilent (G1312A Binary Pump Serial#DE43616357, G1329A ALS autosampler Serial#DE43613/10, G1315A DAD Serial#DE72002469). As stationary phase the Hypersil APS-2 column (125 × 4 mm, 3 μm particle size) of Thermo Fisher Scientific Inc. (Waltham, MA, United States) was used, heated to 40°C. As mobile phase an isocratic flow of 1 mL⋅min^–1^ 68% Acetonitrile (ACN) (solvent B) and 32% phosphate buffer (0.62 g⋅L^–1^ KH_2_PO_4_ and 0.38 g⋅L^–1^ Na_2_HPO_4_⋅2H_2_O) (solvent A) was applied. 40 μL of each sample was injected and separated in a 10 min run. Detection of acarbose was carried out with a DAD detector at 210 nm (Reference 360 nm) and quantified from the peak areas of a calibration curve.

### Substrate Analytics

The supernatant was centrifuged (20,000 g, 2 min), diluted in di-deionized water and transferred into HPLC vials. The analysis was performed in the *smartline* HPLC system of Knauer (Berlin, Germany). As stationary phase the column VA300/7.8 Nucleogel sugar 810-H (300 mm) and precolumn CC 30/4 Nucleogel Sugar 810-H (3 × 4 mm) of Macherey-Nagel (Düren, Germany) was used, heated to 72°C. As mobile phase an isocratic flow of 0.8 mL⋅min^–1^ 2.5 mM H_2_SO_4_ was applied. Twenty microliter of each sample was injected and separated in a 25 min run. Detection of substrates was carried out with a refraction index detector of ERC GmbH (Riemerling, Germany) and quantified from the peak areas of the calibration curve of standards (1–20 g⋅L^–1^). The chromatograms were evaluated with the Chromastar software (SCPA GmbH, Weyhe, Germany).

### Recombinant DNA Work

#### Deletion of the Gene *amlE* (*ACSP50_2474*) and *amlR* (*ACSP50_2475*) by CRISPR/Cas9 Technique

For the creation of deletion mutants of the genes *amlE* (*ACSP50_2474*) and *amlR* (*ACSP50_2475*) by CRISPR/Cas9 technique, the plasmid pCRISPomyces-2 (Cobb et al., 2015) was used according to a protocol of Wolf et al. (2016). The spacer and their reverse complement were ordered at metabion GmbH (Steinkirchen, Germany) as oligonucleotides with overlap (Supplementary Table S1). The oligonucleotides were annealed to double-strands and assembled with the plasmid by *Bsa*I-Golden Gate Assembly (Engler et al., 2008) according to the protocol of Cobb et al. (2015). For repair of the Cas9-induced double-strand break, a DNA template was cloned into the vector by Gibson Assembly (Gibson et al., 2009). As DNA template, flanking sequences up- and downstream of the target gene (each round about 1 kB) were amplified by PCR with the Phusion^®^ High-Fidelity PCR Master Mix with GC Buffer (NEB, Ipswich, MA, United States) (according to the protocol of Cobb et al., 2015; Wolf et al., 2016). The primer sequences are shown in Supplementary Table S1. The reaction mix was transferred to *E. coli* DH5αMCR by chemical transformation according to a protocol of Beyer et al. (2015). Growth and selection of *E. coli* was performed by plating them on LB-media with 15 g⋅L^–1^ agar-agar (Carl Roth, GmbH&Co.KG, Karlsruhe, Germany) supplemented with 50 mg⋅L^–1^ apramycin-sulfate and incubation for 10–14 h at 37°C. Positive colonies were tested by PCR and gel-electrophoresis, and verified by Sanger sequencing of the vector by our in-house sequencing core facility (primer sequences for PCR: for: 5′-GGCGTTCCTGCAATTCTTAG-3′, rev: 5′-TCGCCACCTCTGACTTGAGC-3′). Cloning was planned and simulated by use of the software Clone Manager 9 (Scientific & Educational Software, Denver, CO, United States) and SnapGene 4.3 (GSL Biotech LLC, Chicago, IL, United States).

#### Conjugal Transfer to *Actinoplanes* sp. SE50/110 and Plasmid Curing

Conjugation was performed with *E. coli* ET12567/pUZ8002 (Kieser et al., 2000) as donor strain and competent *Actinoplanes* sp. SE50/110 cells according to a protocol of Schaffert et al. (2019).

Plasmid curing was performed according to the protocol of Wolf et al. (2016). Exconjugants were tested for the deletion by PCR. The PCR-fragment was excised from the gel and sequenced by our in-house Sanger sequencing core facility. Additionally, genomic DNA of the deletion mutant was sequenced by the MinION^®^ of Oxford Nanopore (Oxford, United Kingdom) to exclude off-target effects by the CRISPR/Cas9 technique. For this, genomic DNA of an NBS-grown culture was isolated with the NucleoSpin^®^ Microbial DNA Kit (Macherey-Nagel, Düren, Germany) and a library prepared with help of the 1D Genomic DNA by ligation-Kit (Oxford Nanopore, Oxford, United Kingdom).

### RNA Work

#### Sampling and RNA Isolation

For transcriptome analysis, 2 × 1 mL samples from *Actinoplanes* cultures were taken during growth phase, separated from the supernatant by centrifugation (10 s) and snap-frozen in liquid nitrogen. Pellets were stored at −80°C until further processing. Cell disruption, RNA isolation and digestion of DNA from frozen cell pellets were performed by use of 2 mL lysing matrix tubes (0.1 mm spherical silica beads, MP Biomedicals, Santa Ana, California, United States) and NucleoSpin^®^ RNA Plus kit in combination with rDNAse Set (Macherey-Nagel, Düren, Germany) according to a protocol of Schaffert et al. (2019).

#### Reverse Transcription Quantitative PCR (RT-qPCR)

Reverse transcription quantitative PCR was carried out according to the protocol of Wolf et al. (2017a) by use of the SensiFast SYBR No-Rox One-Step Kit (Bioline, London, United Kingdom) in 96 well lightcycler plates (Sarstedt, Nümbrecht, Germany) in a LightCycler 96 system of Roche (Mannheim, Germany) by use of the software LightCycler^®^ 96 SW 1.1 (Roche, Mannheim, Germany). The relative RNA amount was normalized on total RNA (100 ng) and calculated as 2^–Δ^ ^Cq^. ΔCq was calculated as the difference of the mean Cq in the mutant strain compared to the control strain. The primers are listed in Supplementary Table S2.

#### RNAseq: Library Preparation, Sequencing, and Data Processing

RNA of triplicates of the wildtype and the deletion mutant was isolated from growing cultures and pooled equimolar separately for the wildtype and the deletion mutant (total amount of 2.5 μg RNA each in 26 μL RNase-free water). rRNA was depleted using the Ribo-Zero rRNA Removal Kit for bacteria (Illumina, San Diego, United States). Successful depletion of rRNA was verified by an Agilent RNA 6000 Pico chip in the Bioanalyzer (Agilent, Böblingen, Germany). cDNA libraries were prepared following protocols from Pfeifer-Sancar et al. (2013) and Irla et al. (2015) by use of the TruSeq stranded mRNA kit (Illumina, San Diego, CA, United States). The libraries were quantified by a DNA High Sensitivity Assay chip in the Bioanalyzer (Agilent, Böblingen, Germany) and sequenced on a 2 × 75 nt HiSeq 1500 run (Illumina, San Diego, CA, United States). Sequencing yielded about 10.7 million read pairs for the wildtype library and 10.2 million read pairs for the deletion mutant library, respectively. Raw reads were quality-trimmed using Trimmomatic v0.3.5 (Bolger et al., 2014). Trimmed reads were mapped to the respective reference sequence (GenBank: LT827010.1) using bowtie2 in the paired-end mode (Langmead and Salzberg, 2012), resulting in 20.7 and 19.5 million mappings. ReadXplorer was used for visualization and differential gene expression analysis (Hilker et al., 2014, 2016).

### Assays From Protein Raw Extract

#### Sampling and Sample Preparation

*Actinoplanes* strains were grown in C-Pur minimal medium in a shake flask cultivation, like described above. To allow comparison to the activities in *E. coli* and *Streptomyces* ssp., strains were grown in the complex medium NBS supplemented with 79 g⋅L^–1^ glucose-monohydrate instead 11 g⋅L^–1^ (see above).

2.5 mL of each culture was harvested, washed twice in wash buffer (0.1 M Tris (pH 7.4), 20 mM KCl, 5 mM MgSO_4_), resuspended in 0.7 mL resuspending buffer (0.1 M Tris (pH 7.4), 20 mM KCl, 5 mM MgSO_4_, 0.1 mM EDTA, 1.5 mM DTT, 10% (v/v) glycerol) and transferred to a screw cap tube containing zirconia/silica micro beads (Bio Spec Products Inc., Bartlesville, United States) of the sizes 0.1 and 0.05 mm. Cells were disrupted in a homogenizer (FastPrep FP120, Thermo Fisher Scientific, Waltham, MA, United States) for three times 20 s at speed setting 6.5 and 5 min on ice in between. After centrifugation (10 min at 4°C), the lysate was transferred to a Chromabond^®^ column Easy (Macherey Nagel, Düren, Germany) to get rid of residual carbohydrates in the supernatant. Before application of the sample, the columns were pre-washed with 3 mL methanol followed by 3 mL water to adjust binding conditions. The flow-through (r. a. 500 μL) was transferred into a new reaction tube and 2 mL of the resuspending buffer was added.

Total protein quantification was carried by a Bradford assay (Roti^®^-Nanoquant, Carl Roth GmbH&Co.KG, Karlsruhe, Germany). The samples were measured in a 200 μL assay in a multi titer plate (flat-bottom Nunc^TM^ 96-Well Polystyrene Plates of Thermo Fisher Scientific, Waltham, MA, United States) in the Tecan reader Infinite M200 (Ref 30016056, Tecan Group AG, Männedorf, Schweiz) according to the manufacturer’s protocol. The software i-control TM Microplate Reader was used for planning and execution of the experiments (Tecan Group AG, Männedorf, Schweiz). BSA was used as standard for a calibration curve ranging from 1 to 100 μg⋅mL^–1^. To achieve similar assay conditions, the samples were adjusted to similar protein amounts, which was controlled by a second Bradford assay.

#### Assay Procedure

Enzyme assays from protein raw extracts were carried out following the protocol of Seibold et al. (2009) with adjusted conditions for *Actinoplanes* sp. SE50/110.

In the hydrolase assay, released glucose was phosphorylated by the activity of a hexokinase to glucose-6P, which is the substrate of a glucose-6P-dehydrogenase. By oxidation to 6-phosphogluconolacton, NADP is reduced to NADPH. NADPH has an absorption maximum at 340 nm. The peak intensity at 340 nm correlates to the amount of released glucose in the sample.

A reaction buffer was prepared consisting of 40 μL 5x phosphate buffer (50 mM potassium phosphate, pH 7.0), 20 μL 10x MgCl_2_-solution (250 mM), 4 μL NADP (100 mM, Carl Roth GmbH, Karlsruhe, Germany), 4 μL ATP (100 mM, Thermo Fisher Scientific, Waltham, MA, United States), 2 U hexokinase from *Saccharomyces cerevisiae* (Sigma-Aldrich, St. Louis, MO, United States), 2 U glucose-6-P-dehydrogenase (AlfaAesar, Haverhill, MA, United States) adjusted to a total volume of 90 μL by addition of ddH_2_O. 10 μL 100 mM sugar (maltose, trehalose maltotriose, maltoheptaose or maltodextrin) or 10 μL ddH_2_O (blank) were added. The reaction was started after addition of 100 μL of the sample. The release of glucose was measured by the formation of NADPH from NADP at 340 nm at 30°C for 3 h.

Phosphorolytic activity was monitored by glucose-1P-release from the activity of putative maltophosphorylases. For this, glucose-1P is converted to glucose-6P by the activity of a phosphoglucomutase. Similar to the hydrolase assay, glucose-6P serves as substrate for a glucose-6P-dehydrogenase, which leads to the reduction of NADP to NADPH.

A reaction buffer was prepared, which consisted of 40 μL 5x phosphate buffer (50 mM potassium phosphate, pH 7.0), 20 μL 10x MgCl_2_-solution (250 mM), 4 μL NADP (100 mM, Carl Roth GmbH, Karlsruhe, Germany), 2 U phosphoglucomutase (Sigma-Aldrich, St. Louis, United States), 2 U glucose-6-P-dehydrogenase (AlfaAesar, Haverhill, MA, United States) adjusted to a total volume of 90 μL by addition of ddH_2_O according to Seibold et al. (2009). Ten microliter 100 mM maltoheptaose or maltodextrins, or 10 μL ddH_2_O (blank) were added. The reaction was started after addition of 100 μL of the sample. The release of glucose-1P was measured by the formation of NADPH from NADP at 340 nm at 30°C for 3 h.

It was not possible to monitor transglycosidase activity, as the activity is covered by the hydrolytic activity.

Each assay was performed by use of three biological replicates, measured in two technical replicates and individual blank for each sample, which was subtracted from the mean value of both technical replicates. The reactions were carried out in a multi titer plate (flat-bottom Nunc^TM^ 96-Well Polystyrene Plates of Thermo Scientific, Waltham, MA, United States) in the Tecan reader Infinite M200 (Ref 30016056, Tecan Group AG, Männedorf, Schweiz).

## Results

### Studies of the Maltose/Maltodextrin Metabolism in *Actinoplanes* sp. SE50/110 With Special Regard to the *amlE-amlR* Gene Arrangement

#### Conserved Domains of the Genes of the *aml* Gene Cluster and Their Genomic Organization in *Actinoplanes* sp. SE50/110 and in Related Species

**FIGURE 1:**
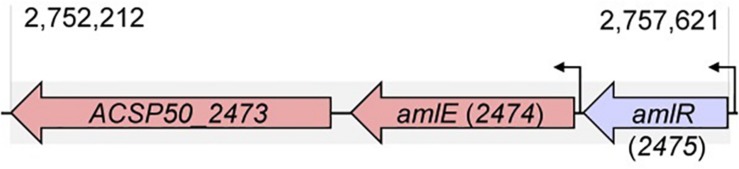
Position of *amlE* in the genome of *Actinoplanes* sp. SE50/110. The *aml* gene cluster consists of genes encoding a PurR/LacI-type transcriptional regulator AmlR (*ACSP50_2475)*, the maltase AmlE (*ACSP50_2474*) and an uncharacterized protein with diguanylate cyclase and phosphodiesterase domain (*ACSP50_2473*).

*AmlE* is localized in a gene cluster together with two further genes (Figure 1): A gene upstream (*ACSP50_2475*), which is oriented in a head-to-tail orientation and encodes a PurR/LacI-like transcriptional regulator, and a gene downstream (*ACSP50_2473*) encoding an uncharacterized protein with diguanylate cyclase and phosphodiesterase domain.

Like ACSP50_2475, which we name *Actinoplanes* maltase Enzyme regulator AmlR, PurR/LacI-type regulators are part of the LacI/GalR regulator family. Members of this regulator family are commonly repressors with N-terminal HTH-motif and C-terminal ligand binding domain with similarity to periplasmic sugar-binding proteins (Fukami-Kobayashi et al., 2003). Nearly 90% of this regulator family are local transcriptional regulators of the sugar metabolism (Ravcheev et al., 2014). According to the functional domain search of the NCBI (Marchler-Bauer et al., 2015, 2017), the C-terminal periplasmic binding protein of AmlR can be classified as type 1 periplasmic binding fold superfamily (data not shown). Members of this family typically bind monosaccharides, which leads to changes of the DNA binding activity of the repressor domain.

ACSP50_2473 is probably involved in the cyclic diguanylate (c-di-GMP) metabolism, which is a universal second messenger used in signal-transduction (Römling et al., 2013). A conserved domain search (Marchler-Bauer et al., 2015, 2017) of ACSP50_2473 shows, that its gene product contains a conserved GGDEF (Gly-Gly-Asp-Glu-Phe)-domain, which is known as active site of diguanylate cyclases (DGCs) (data not shown). Additionally, also an EAL (Glu-Ala-Leu)-domain is found, known as active site of phosphodiesterases (PDEs) (data not shown). Whereas proteins with GGDEF domain were described to catalyze cyclic diguanylate from two molecules GTP (Römling et al., 2013; Tschowri, 2016), proteins with EAL domain are described to be involved in its degradation: By attacking one ester bond of the circular dinucleotide, a linear dinucleotide (5′-phosphoguanylyl-(3′–5′)-guanosine, short: pGpG) is created, which was suspected to act as signaling molecule as well (reviewed by Römling et al., 2013; Tschowri, 2016). According to TMHMM Server v. 2.0, a tool for prediction of transmembrane helices in proteins (Krogh et al., 2001), ACSP50_2473 contains an N-terminal transmembrane domain (data not shown).

**FIGURE 2:**
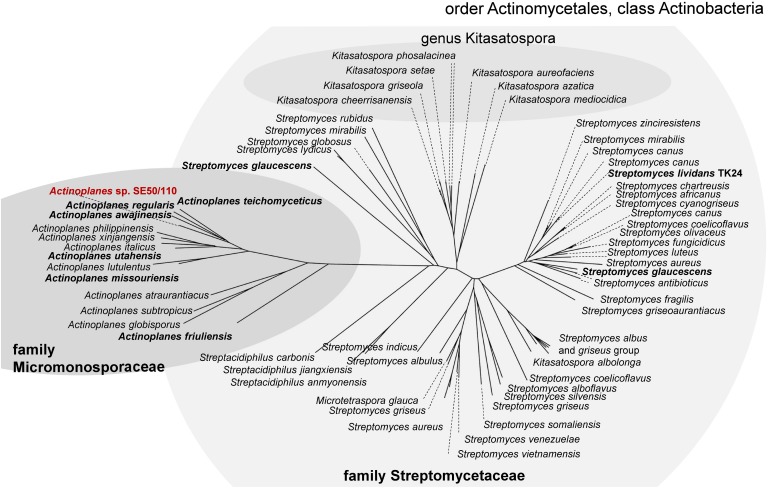
PhiBlast-analysis against the prokaryotic refseq-database of the NCBI (Altschul et al., 1997) displays a high distribution of glycoside hydrolase family 13 proteins with sequence homology to *amlE* within the class of Actinobacteria.

AmlE is a glycoside hydrolase family 13 protein. PhiBlast-analysis against the prokaryotic refseq-protein database of the NCBI (Altschul et al., 1997, 2005) displays several glycoside hydrolase family 13 proteins within the family Micromonosporaceae with high sequence identity to AmlE (sequence identities between 76-90% and positives of 100%) (Figure 2). Homologs also occur in related species of the family Streptomycetaceae (sequence identities between 64-71% and positives between 93-100%) (Figure 2). This might indicate for a gene duplication of AmlE within this taxon.

**FIGURE 3:**
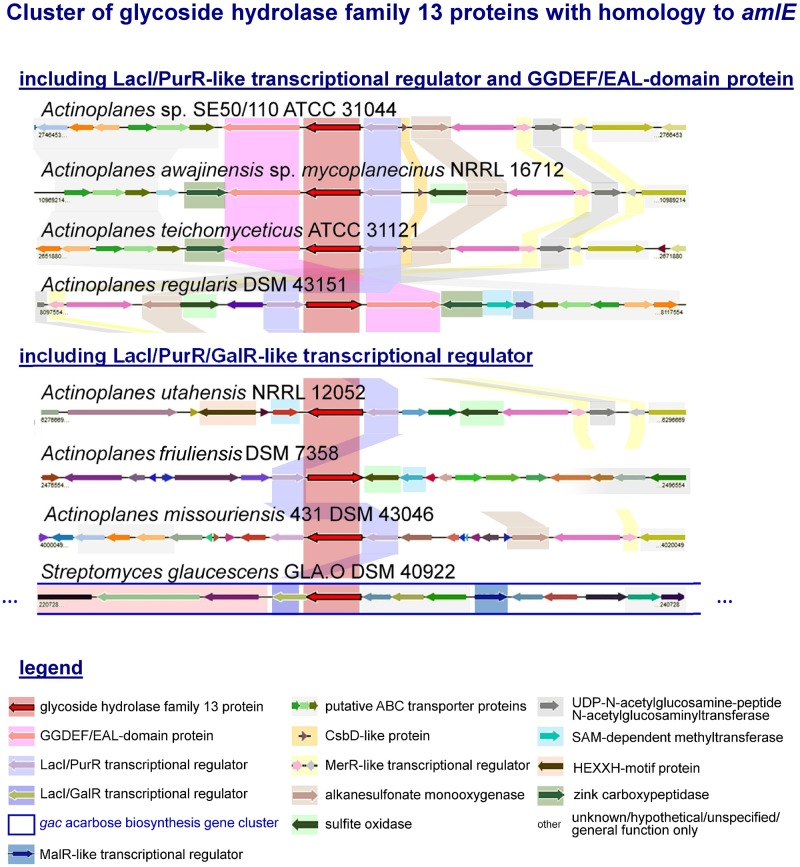
Cluster of glycoside hydrolase family 13 proteins with homology to *amlE* from *Actinoplanes* sp. SE50/110 and their genomic environment according to an analysis with the software EDGAR 2.0. Orthologous genes are highlighted in color. Most environments include genes encoding for putative ABC transport proteins, alkanesulfonate monooxygenase, UDP-N-acetylglucosamine-peptide N-acetylglucosaminyltransferase, which is surrounded by one or two tandem-oriented MerR-like transcriptional regulators. In four cases, a GGDEF-EAL domain containing gene was found, three of which contain a gene with CsbD-like domain divergently placed from the LacI/PurR transcriptional regulator. Most environments contain a zinc-carboxypeptidase or a HEXXH-motif protein, which is missing in SE50/110. A gene encoding for a sulfite oxidase occurs in 4 cases, of which the one in *A. utahensis* is localized in a head-to-head orientation to a sensor-histidine kinase (gene not specially colored). *A. regularis* furthermore encodes a SAM-dependent methyltransferase and TetR-transcriptional regulator in the direct vicinity of the *amlE-amlR* gene arrangement. The environment of *amlE/amlR* homologs in *A. friuliensis* includes hypothetical genes or genes of unknown function, some of which contain a HTH- and oxidoreductase domain. In *S. glaucescens* GLA.O the *amlE*-homolog is part of the *gac* acarbose biosynthesis gene cluster.

By EDGAR 2.0, a software platform for comparative genomics (Blom et al., 2016), orthologous genes of AmlE were searched in the genome of related species to see, if organization of *amlE-amlR-ACSP50_2473* was conserved within the family Micromonosporacea. We found a similar gene cluster including a PurR/LacI-like transcriptional regulator and a GGDEF-EAL-domain protein in *Actinoplanes awajinensis* sp. *mycoplanecinus* NRRL 16712, *Actinoplanes teichomyceticus* ATCC 31121 and *Actinoplanes regularis* DSM 43151 (Figure 3).

The environments of these gene clusters include genes coding for putative ABC transport proteins, an alkanesulfonate monooxygenase of sulfur metabolism, an UDP-N-acetylglucosamine-peptide N-acetylglucosaminyltransferase putatively involved in protein glycosylation, which is surrounded by genes encoding two tandem-oriented MerR-like transcriptional regulators (Figure 3). With exception of *A. regularis*, a small protein with CsbD-like domain is annotated in direct vicinity to the *amlE-amlR* gene arrangement (Figure 3). CsbD is a bacterial general stress response protein of unknown function, whose expression is mediated by Sigma-B, an alternative sigma factor (Prágai and Harwood, 2002). *A. awajinensis, A. teichomyceticus*, and *A. regularis* include a gene coding for a zinc-carboxypeptidase, which is missing in SE50/110. *A. awajinensis* and *A. regularis* additionally include a sulfite oxidase of sulfur metabolism. *A. regularis* furthermore encodes a SAM-dependent methyltransferase and TetR-like transcriptional regulator in the direct vicinity of the *amlE-amlR* gene arrangement.

In *A. utahensis* NRRL 12052, *A. friuliensis* DSM 43151, *A. missouriensis* 431 DSM 43046 and *Streptomyces glaucescens* GLA.O DSM 40922, the putative *amlE-*homolog occurs together with a PurR/LacI/GalR-like transcriptional regulator gene, but not with a GGDEF-EAL-domain gene (Figure 3). For *A. utahensis* and *A. missouriensis* a similar genomic environment like described above was found (Figure 3). The environment of *A. friuliensis* differs substantially: Here, several hypothetical genes or genes of unknown function are located, some of which contain unspecific annotations as HTH-domain protein or oxidoreductase.

Interestingly, the *amlE*-homolog of *S. glaucescens* GLA.O was identified as part of the *gac* acarbose biosynthesis gene cluster (Figure 3). One further *amlE*-homolog is encoded in *S. glaucescens* GLA.O: Here, the environment is similar to the ones from *S. lividans* TK24 and *S. coelicolor* A3(2), in which the AmlE-homolog is localized together with further genes of the sugar metabolism but not together with a LacI/PurR/GalR-type regulator gene (data not shown).

#### Bioinformatic Analysis of the Maltose/Maltodextrin Metabolism and Investigation of a Sugar-Dependent Transcription Profile

In order of making first steps in unraveling of the maltose/maltodextrin metabolism, we searched for putative gene homologs from the models of the well-studied organisms *E. coli* and C. *glutamicum* by BlastP-analysis (Altschul et al., 1997, 2005). We found gene homologs of the amylomaltase MalQ, the glucosidases MalZ and further genes of the maltose/maltodextrin/glycogen metabolism (Supplementary Table S3 and Supplementary Figure S1). Interestingly, a MalP-homolog could not be identified in the genome of *Actinoplanes* sp. SE50/110. Also, BlastP-analysis against the nr database of the taxonomic group Actinomycetales shows hits with highest identities and positives (49% identities, 64% positives) for genes annotated as glycogen phosphorylase (GlgP), but no hits for genes annotated as maltodextrin phosphorylase (MalP) were found (data not shown). This may indicate for a lack of MalP-phosphorylase within this taxon.

An AmlE-like protein has not been described for the model organism *E. coli* or *C. glutamicum.* According to that, BlastP analyses only display hits with week sequence homology to enzymes annotated as glycosyl-transferase (*C. glutamicum*: 29% identities, 43% positives) or alpha-phosphotrehalase (*E. coli*: 36% identities, 50% positives) (Supplementary Table S3). Likewise, also the maltase MalL from *B. subtilis* displays only week sequence similarity to AmlE from *A.* sp. SE50/110 (30% identities, 46% similarities). No further putative homologs of the *B. subtilis* maltose/maltodextrin system were found in *A.* sp. SE50/110. We therefore assume, that the maltose/maltodextrin system and the role of MalL/AmlE have evolved differently in *B. subtilis* and *A.* sp. SE50/110.

Further putative *amlE* homologs exist in *A.* sp. SE50/110, namely *ACSP50_1177, ACSP50_6814* and *ACSP50_6830*, annotated as α-glycosidases (Supplementary Table S3).

**FIGURE 4:**
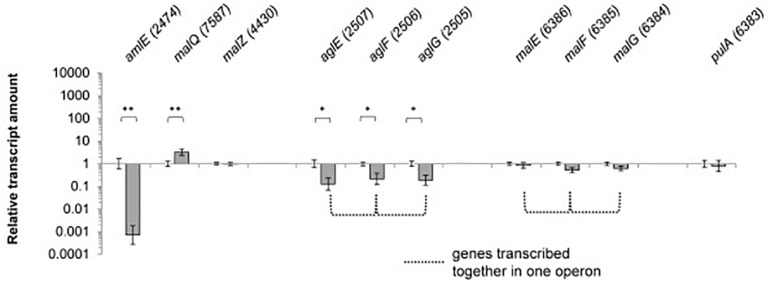
Relative transcript amounts of the genes of the maltose/maltodextrin metabolism and transport, when grown on glucose compared to a maltose-grown culture of *Actinoplanes* sp. SE50/110 (relative transcript amount of maltose set to 1). Significance of differential transcription was calculated by a two-sided *t*-test. Asterisks indicate the significance value (^∗^*p* < α = 5%, ^∗∗^*p* < α = 1%) (calculated *p*-values: *amlE:* 0.001533, *malQ:* 0.004633, *malZ:* 0.05057, *aglE*: 0.01164, *aglF*: 0.03542, *aglG*: 0.01507, *malE:* 0.3132, *malF:* 0.07183, *malG:* 0.1593, *pulA:* 0.5535).

Since expression of genes of the maltose/maltodextrin metabolism and transporter systems has been described to be sugar-dependent in *E. coli, C. glutamicum* and *B. subtilis*, we analyzed the expression of the identified gene homologs in SE50/110 on glucose compared to a maltose-grown culture (Supplementary Figure S2). The genes *amlE* and *aglEFG* were significant differentially expressed, displaying reduced expression on glucose compared to maltose [log_2_(fold-change]: *amlE*: 1,390, *aglE:* 7.9, *aglF*: 4.6, *aglG*: 5.3) (Figure 4 and Supplementary Figure S2). For the gene *amlE*, glucose-repression was additionally shown in mixtures of glucose and maltose (Supplementary Figure S3). On high molecular maltodextrins, expression of *amlE* was similar compared to maltose (Supplementary Figure S3).

Interestingly, the central genes of the maltose/maltodextrin metabolism *malZ* and *malQ* as well as the putative maltose transporter genes *malEFG* were not found to be glucose-repressed (Figure 4). For the amylomaltase MalQ, even enhanced transcription was monitored on glucose compared to maltose [log_2_(fold-change): 3.3].

### Importance and Function of the Maltase AmlE

#### Growth Phenotype of the Deletion Mutant Δ*amlE* on Different Carbon Sources

**FIGURE 5:**
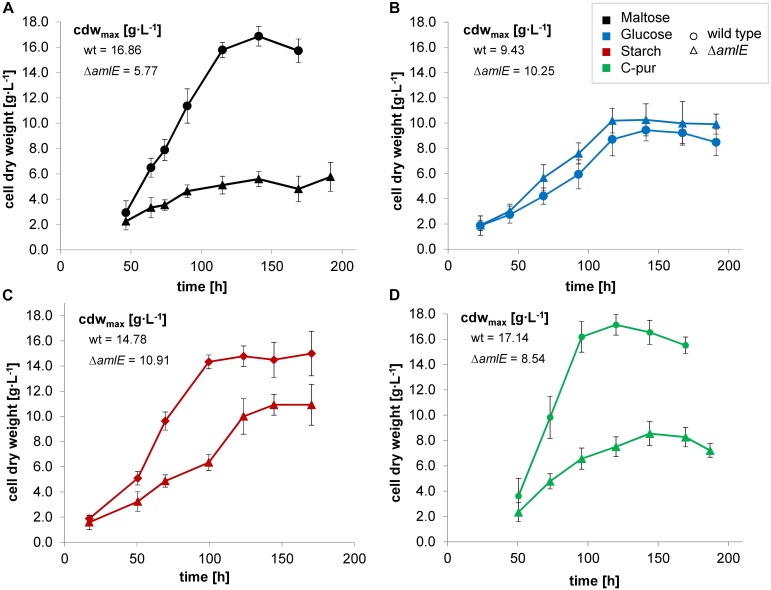
Growth of the wild type (Δ) and the deletion mutant Δ*amlE* (Δ) of *Actinoplanes* sp. SE50/110 in minimal medium supplemented with different carbon sources: maltose (**A**, black), glucose (**B**, blue), starch (**C**, red) and C-Pur (**D**, green). The deletion mutant Δ*amlE* displays strong growth inhibitions on maltose (**A**, number of biological replicates n: n_Δ_*_*amlE*_* = 4, n_wt_ = 5), reduced growth on starch (**C**, number of biological replicates n: n_Δ_*_*amlE*_* = 3, n_wt_ = 4), and nearly the same growth on glucose (**B**, number of biological replicates n: n_Δ_*_*amlE*_* = 4, n_wt_ = 4) compared to the wild type. In C-Pur, Δ*amlE* reaches half of the final cell dry weight compared to the wild type (**D**, number of biological replicates n: n_Δ__amlE_ = 4, n_wt_ = 4). The averages of the mean maximal cell dry weights (cdw_max_) of the deletion mutant Δ*amlE* and the wild type were compared by a two-sided t-test [*p*-values: maltose **(A)**: 0.0003538, glucose **(B)**: 0.2021, starch **(C)**: 0.02508, C-pur **(D)**: 8.732e-06].

A deletion mutant of the gene *amlE* was created by CRIPSR/Cas9 technique. The deletion mutant Δ*amlE* was cultivated in minimal medium on different carbon sources. Compared to the wild type, it displays normal to slightly enhanced growth on glucose (Figure 5B, ▲) and strong growth retardation on maltose (Figure 5A, 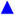). According to substrate analytics, maltose is not metabolized in the deletion mutant Δ*amlE*, whereas glucose is consumed at wild type level (Supplementary Figures S4A,B). The growth of Δ*amlE* was better on starch and C-pur compared to growth on maltose (Figure 5C, 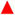 and Figure 5D, 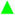). C-Pur (*Cerestar* 01908) is a sugar product from the degradation of starch, which consists mainly of maltose (43%) and maltotriose (40%) and higher maltodextrins (probably maltotetraose, 16%) according to our substrate analyses (data not shown). Here, Δ*amlE* reaches half of the maximal cell dry weight compared to the wild type (Figure 5D, 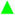). On the complex carbon source starch, Δ*amlE* does not reach the wild type level (Figure 5C, 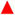).

To rule out direct inhibitory effects by maltose, mixtures of maltose and glucose minimal medium in a ratio of 50:50 and 90:10 were prepared. In a shake flask cultivation, the deletion mutant Δ*amlE* displayed slightly reduced growth compared to the wild type, but the growth phenotype was significantly improved approaching approximately 80% of the final cell dry weight of the wild type under both conditions (Supplementary Figure S5). In mixtures of glucose and maltose, the deletion mutant Δ*amlE* is able to form acarbose in dependence on the growth phenotype (Supplementary Figure S6). Since acarbose is a growth-associated product (Wendler et al., 2014; Wolf et al., 2017a) and the growth phenotype was not completely restored in mixtures of maltose and glucose, the final acarbose yields were reduced reaching r. a. 70% of the wild type. This, however, was led back to growth effects. The composition and the relation of the different acarviosyl-metabolites formed by Δ*amlE* is comparable to the one from the wild type: In both strains, acarviosyl-maltose is the main component, when growing on maltose as carbon sources, whereas acarviosyl-glucose, acarviosyl-triose and acarviosyl-tetraose are formed to a lesser extent (data not shown), similar to findings from Wendler et al. (2014). In mixtures of maltose and glucose, the porportion of acarviosyl-glucose is higher, but similar in the wild type and the deletion mutant (data not shown). Taken all together, no direct connection to the acarviosyl-metabolite synthesis was encountered for AmlE.

#### Enzyme Assays Confirm a Central Role in the Degradation of Maltose and Lack of MalP in *Actinoplanes* sp. SE50/110

To evaluate the enzymatic spectrum of AmlE, activity assays from protein raw extracts of the deletion mutant Δ*amlE* and the wild type were carried out following a protocol of Seibold et al. (2009). Due to the fact, that growth of Δ*amlE* is impaired on maltose (section Growth Phenotype of the Deletion Mutant *ΔamlE* on Different Carbon Sources) and expression of *amlE* is significantly down-regulated in presence of glucose (Supplementary Figure S3A), C-Pur was used as carbon source, growing on which the gene *amlE* is highly expressed in the wild type (Supplementary Figure S3A). Starch was not suitable for conducting of the assay, due to the turbidity of the medium. Similar to previous cultivations (Figure 5D), Δ*amlE* reached half of the maximal cell dry weight of the wild type (Supplementary Figure S7). The assay was performed during early growth phase. Due to the growth differences, the protein raw extracts were adjusted to similar protein amounts.

**FIGURE 6:**
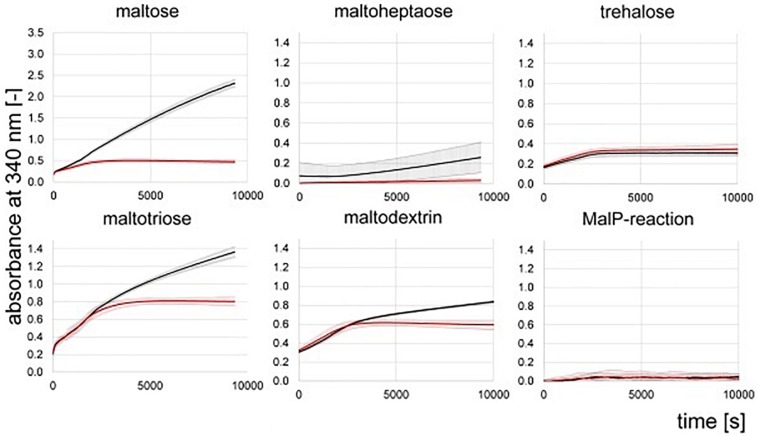
NADPH-release from hydrolytic enzyme assays measured by absorbance at 340 nm. Shown are the means and standard deviations of triplicates of the wild type (black) and the deletion mutant Δ*amlE* (red). Samples for the assay were taken during growth phase after 47 h of cultivation (Supplementary Figure S7).

Hydrolase activity was detected for maltose, maltotriose and maltodextrins in the protein raw extracts of the wild type of *Actinoplanes* sp. SE50/110 (Figure 6). Only spurious hydrolase activity was determined for the deletion mutant Δ*amlE* on maltose (Figure 6). Maltotriose and maltodextrin were hydrolyzed at similar rates between 0 and 2000 s compared to the wild type (Figure 6). Afterward, hydrolase activity decelerates for maltotriose and maltodextrin in the protein raw extract of Δ*amlE*, probably due to release of maltose from maltotriose and maltodextrin, which does not serve as substrate. Concerning trehalose, hydrolase activity does not differ in the wild type and the deletion mutant Δ*amlE*, but it was considerably lower compared to α-1,4-linked sugars (Figure 6). Maltoheptaose was not hydrolyzed (Figure 6). In a different experimental approach, also hydrolase activity of the α-1,6 linked isomaltose was tested, but no differences were detected for the wild type and Δ*amlE* (Supplementary Figure S8). Transglycosidase activity (MalQ) could not be monitored with this kind of assay, as it is masked by the hydrolytic activity.

**FIGURE 7:**
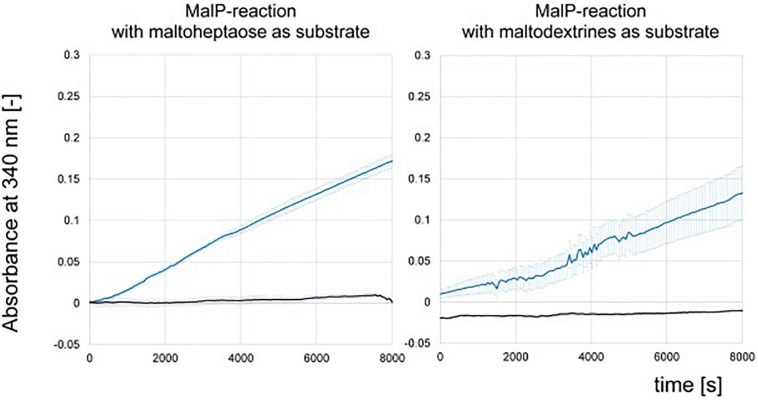
NADPH-release from MalP-enzyme assays measured by absorbance at 340 nm of protein raw extract from *Escherichia coli* DH5α (blue) and *Actinoplanes* sp. SE50/110 (black). Shown are the means and standard deviations of triplicates.

We could not detect phosphorylase activity in the protein raw extracts of the wild type and the deletion mutant Δ*amlE* by use of the substrate maltoheptaose (Figure 6). For further validation, protein raw extracts from cells of the wild type of *Actinoplanes* sp. SE50/110 and *E. coli* DH5α were prepared, both of which were grown in the complex medium NBS. To prove quality of the protein raw extracts and exclude inactivation of the enzymatic function by denaturation during the extraction process, hydrolytic activity was measured positive for all extracts in advance (data not shown). The MalP-assay was performed by use of the substrates maltoheptaose and a mixture of maltodextrins. Indeed, no MalP-activity was detected in the wild type of *Actinoplanes* sp. SE50/110, whereas strong phosphorolytic activity was shown for *E. coli* on both substrates (Figure 7).

Similar results were obtained from crude extracts of *S. lividans* TK23, a plasmid-free derivative of *S. lividans* 66 (Kieser et al., 2000), *S. coelicolor* A3(2) M145 (ATCC BAA-471), and *S. glaucescens* GLA.O (DSM 40922): Here, no MalP activity was measured neither (Supplementary Figure S9), which is in accordance with the sequence similarity analyses described above.

### The PurR/LacI-Type Regulator AmlR Is the Local Transcriptional Repressor of the *aml* Operon

The PurR/LacI-type transcriptional regulator AmlR (ACSP50*_*2475) of the LacI/GalR regulator family, is a promising candidate for the regulation of the sugar-dependent expression due to its position upstream of *amlE* (Figure 1).

**FIGURE 8:**
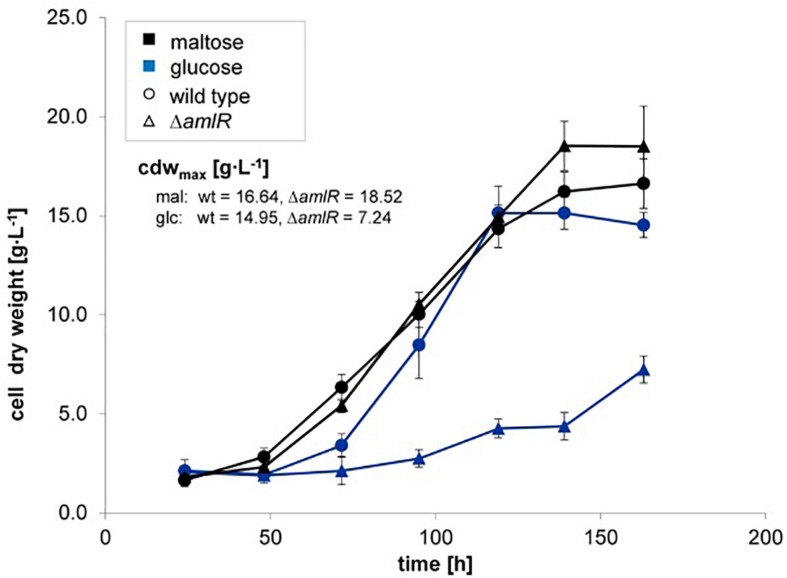
Growth of wild type (⚫) and the deletion mutant ▲*amlR* (Δ) of *Actinoplanes* sp. SE50/110 in minimal medium supplemented with the carbon source glucose (dark-blue, number of biological replicates n: n_Δ_*_*amlR*_* = 3, n_wt_ = 3) or maltose (black, number of biological replicates n: n_Δa_*_*mlR*_* = 3, n_wt_ = 5). Shown are the means and standard deviations of cell dry weights of a shake flask cultivation. Comparison between the deletion mutant Δ*amlR* with the wild type displays similar growth behavior on maltose (▲) and strong growth inhibitions on glucose (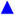).

A deletion mutant was created by CRISPR/Cas9 technique, further on referred as Δ*amlR.* The regulator mutant displays normal growth on maltose (Figure 8, ▲) and severe growth inhibitions on glucose as carbon source (Figure 8, 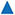). This growth phenotype could be restored in mixtures of maltose and glucose (Supplementary Figure S10). The acarbose formation in Δ*amlR* on maltose minimal medium does not differ significantly from the wild type (*p*-value of a two-sided *t*-test = 0.0965) (Supplementary Figure S11).

**FIGURE 9:**
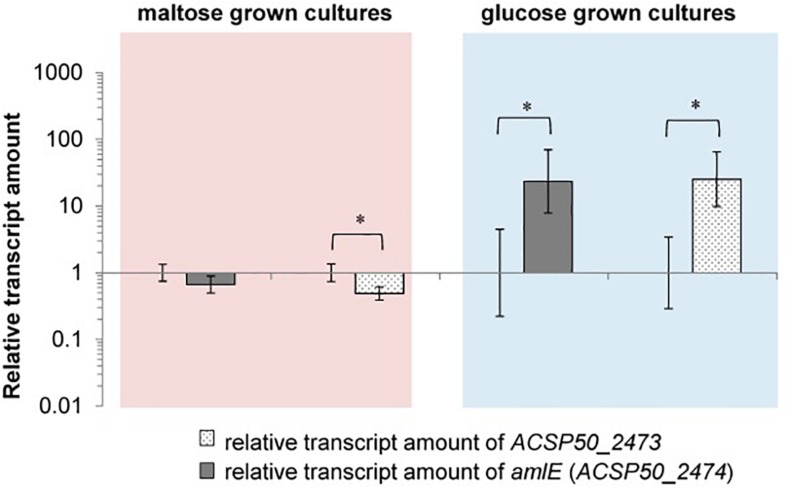
Relative transcription of *amlE (ACSP50_2474)* and *ACSP50_2473* in the deletion mutant Δ*amlR* compared to the wild type of *Actinoplanes* sp. SE50/110, when cells are grown on maltose or glucose. In maltose minimal medium the expression of the *aml* operon is reduced in Δ*amlR* compared to the wild type [log_2_(fold-change) of 0.67 (*amlE*) and 0.49 (*ACSP50_2473*)]. In glucose minimal medium the expression is approximately 24-fold stronger in Δ*amlR* compared to the wild type [log_2_(fold-change) of 23.43 (*amlE*) and 25.24 (*ACSP50_2473*)]. All of these differences were tested by a two-sided *t*-test [*p*-values (from **left** to **right**) = 0.0749, 0.0182, 0.0313, 0.0150]. The asterisk indicates the significance value of *p* < α = 5%.

To evaluate a regulatory effect on the *aml* operon, RNA from the deletion mutant Δ*amlR* and the wild type was isolated, and the relative transcript amounts of *amlE* (*ACSP50_2474*) and *ACSP50_2473* measured by RT-qPCR. Significant increased transcript amounts were detected in absence of the regulator AmlR, when cells were grown on glucose (Figure 9). Both genes – *amlE* and *ACSP50_2473* – were found to be around 23–25-fold stronger transcribed in the regulator mutant. This indicates for a function as transcriptional repressor.

For the maltose-grown culture, the relative transcript amounts of *amlE* are similar in Δ*amlR* compared to the wild type and slightly reduced for *ACSP50_2473*, but only to a small extent (Figure 9).

We also analyzed the transcription of adjacent localized genes. They do not differ from the wild type (Supplementary Figure S14A). Only exception is a single gene (*ACSP50_2471*) in the middle of the transporter operon *ACSP50_2470-2*. This operon is very weakly transcribed on both glucose and maltose minimal medium. Due to this, we assign this result to a technical side-effect caused by the low basis transcription of the operon and low RNA quality, which was reasoned by the poor growth phenotype of Δ*amlR* on glucose.

**FIGURE 10:**
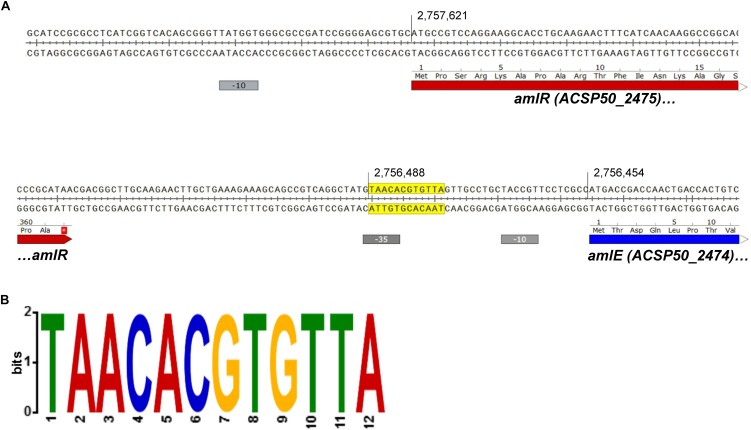
Palindromic region upstream of *amlE* overlapping to the promoter region. **(A)** Position of the palindrome (yellow) within the promoter region of *amlE* (blue and red indicating coding sequences and gray the -10 and -35 hexamers). **(B)** Motif generated and visualized by the MEME suite shown as 2-bit logo (Bailey and Elkan, 1994; Bailey et al., 2009).

The sequence upstream of *amlE* was analyzed by the MEME suite (Bailey and Elkan, 1994; Bailey et al., 2009) in order to find palindromic sequences, that could serve as putative binding sites for the repressor AmlR. One single palindromic region was identified in front of *amlE*, which overlaps with the -35-hexamer of the promoter region (Figure 10).

The palindrome in front of *amlE* meets the expectations of a PurR/LacI-associated binding motif: LacI-type palindromes are commonly located 140–30 bp upstream of the start codon (75%) (Ravcheev et al., 2014). Most of the palindromes are even palindromes, whereas non-canonical palindromes were only found in 4% of all cases (Ravcheev et al., 2014). Most of the even palindromes contain a consensus CG pair in the center (Ravcheev et al., 2014). The identified motif within the -35-region of the promoter of *amlE* is an even palindrome with central CG pair. With 12 nucleotides, the motif is comparatively short. However, palindromic binding sites with only 12 conserved nucleotides have already been described for PurR-like transcriptional regulators by Ravcheev et al. (2014). Variations of the palindrome were scanned against the whole genome by use of the tool FIMO (Grant et al., 2011). The exact sequence displayed in Figure 10B only occurs once in the genome: in front of *amlE.*

To identify further putative targets of the repressor AmlR, additional transcriptome analyses of Δ*amlR* and the wild type grown on glucose minimal medium were carried out: For global pre-screening we used a pooled library for RNAseq (Supplementary File S2) and validated these results for a total of 25 genes by RT-qPCR (Supplementary Figure S14).

Because of growth restraints (Figure 8) and – consequently – poor RNA quality, which complicates transcriptome analyses, the response on RNA-level was highly diverse (Supplementary Figure S12) and various functional classes were affected (Supplementary Figure S13). According to DeSeq2 analyses (Love et al., 2017), a total of 313 genes were significant differentially transcribed (list of differentially transcribed genes in Supplementary File S2). Unexpected for the deletion mutant of a repressor, 207 genes were significantly less and 106 significantly stronger transcribed in Δ*amlR* compared to the wild type (Supplementary Figure S12). They encode transport systems (29), metabolic reactions (86), cellular processes and signaling (33), replication and DNA repair (3) and information storage and processing (31) including 23 putative transcriptional regulators, and further functions (Supplementary Figure S13).

The complex response on transcriptional level and the growth phenotype of Δ*amlR* account for complex, multi-faceted and pleiotropic secondary effects rather than a primary effect by deletion of AmlR. In this context, overexpression of the gene *ACSP50_2473* in the regulator mutant (Figure 9) could play a vital role, since it is probably involved in the metabolism of the global second messenger c-di-GMP.

## Discussion

### Reconstruction of the Maltose/Maltodextrin Metabolism in *Actinoplanes* sp. SE50/110

The disaccharide maltose is the main carbon source in most culturing media of *Actinoplanes* sp. SE50/110, because it serves as energy supplier on the one hand and as key precursor of the acarbose biosynthesis on the other hand. However, little was known about the maltose utilization before.

**FIGURE 11:**
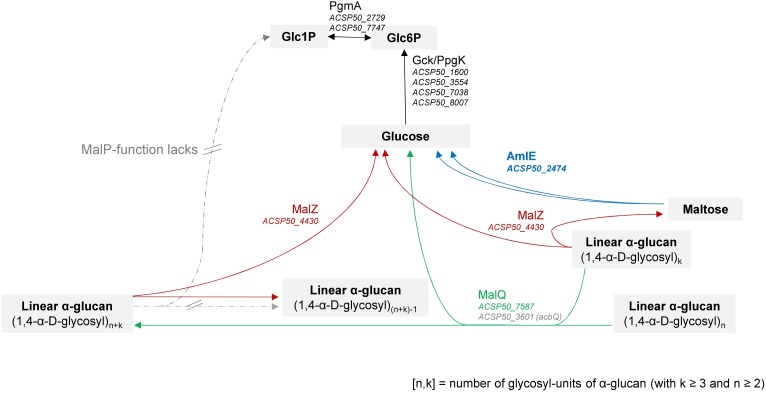
Reconstruction of the maltose/maltodextrin system in *Actinoplanes* sp. SE50/110 according to homology comparison to other microorganisms, like f. e. *Escherichia coli* (Boos and Shuman, 1998; Park et al., 2011) and *Corynebacterium glutamicum* (Seibold, 2008; Seibold et al., 2009). The complete reconstruction and further literature is referenced in Supplementary Figure S1 and Supplementary Table S3.

We reconstructed the maltose/maltodextrin metabolism in *Actinoplanes* sp. SE50/110 by following the models of *E. coli* (Boos and Shuman, 1998; Park et al., 2011), *C. glutamicum* (Seibold et al., 2007, 2009; Seibold and Eikmanns, 2007; Woo et al., 2010) and further microorganisms (Supplementary Figure S1 and Figure 11). By functional prediction of the improved genome annotation of Wolf et al. (2017b) and homology comparison by BlastP analysis (Altschul et al., 1997, 2005), we could assign gene homologs to all described functions with exception of MalP. As neither a MalP-phosphorolytic function could be proven by an *in vitro* assay from protein raw extracts of the wild type, we are convinced, that this function lacks in *Actinoplanes* sp. SE50/110.

Absence of a MalP-function in SE50/110 is striking, since for the models of *E. coli* and *C. glutamicum* both MalQ and MalP have been described to be required for an efficient utilization of maltodextrins of different sizes (Boos and Shuman, 1998; Seibold et al., 2009; Park et al., 2011).

Interestingly, instead of the phosphorolytic MalP-activity, the hydrolytic AmlE-activity turned out to be indispensable for the assimilation of maltose in *Actinoplanes* sp. SE50/110.

AmlE is a maltase, which was reported to be excessively expressed in *A.* sp. SE50/110 when growing on maltose as carbon source (Wendler et al., 2015a). By our assay, we could assign a central role in the assimilation of maltose, as the maltase activity completely lacks in the protein raw extracts of Δ*amlE*. A central function in the hydrolysis of trehalose, isomaltose or higher maltodextrins could not be confirmed by our assay. In accordance to that, severe growth deficiencies were observed for Δ*amlE* on maltose as carbon source. This growth phenotype could be partially restored by supplementation with higher maltodextrins or glucose. It appeared that AmlE is mainly responsible for the degradation of maltose, which cannot be replaced by other glycosidases of the maltose/maltodextrin system. Based on this, we conclude, that the activities of MalQ- and MalZ-homologs are not sufficient to meet the needs of the cell in the deletion mutant Δ*amlE* when growing on maltose. This is in accordance with what we expected from other bacteria: In *E. coli*, it has been reported, that maltose does not serve as substrate of MalQ and MalZ and a minimal length of three glycosyl-residues is required (Boos and Shuman, 1998; Park et al., 2011). Consequently, also putative *amlE* homologs identified in SE50/110 by sequence comparison do not represent true functional homologs of AmlE.

We assume, that in *Actinoplanes* sp. SE50/110, AmlE has a primary function in the maltose assimilation within the maltose/maltodextrin system (Figure 11). This function is irreplaceable, which could be reasoned by the lack of a phosphorolytic function (MalP), due to which assimilation of maltose and maltodextrins depends on the transglycolytic and hydrolytic activities.

We wonder, if the presence of AmlE-homologs in the class Actinomycetales correlates with an absence of a MalP-function. BlastP-analyses against the nr database of the taxonomic group Actinomycetales, reveal, that most of these species encode for a glycogen phosphorylase GlgP rather than a maltodextrin phosphorylase MalP. This might indicate, that further species of this order lack a MalP-function, which was proven for three representative species of the family Streptomycetaceae. Albeit, the maltose/maltodextrin systems of these hosts have – to the best author’s knowledge – not been analyzed yet and therefore need further investigation.

Taken all together, we provide a first model for the maltose/maltodextrin metabolism in *Actinoplanes* sp. SE50/110, in which the lack of MalP and the presence of AmlE seem to be characteristic. A transferability of this model to other representatives of the taxon remains to be examined.

### Occurrence of the *amlE-amlR* Gene Arrangement in the Family Micromonosporaceae and Interconnection With the Acarbose Biosynthesis Gene Cluster

Glycoside hydrolase 13 proteins with homologies to AmlE are widely distributed. In species of the family Micromonosporaceae, *amlE*-homologs were found in an operon together with a PurR/LacI/GalR regulator gene in a genetic environment encoding various functional classes. In contrast, it is localized without regulatory gene and together with further proteins of the sugar-metabolism in species of the family Streptomycetaceae. This indicates that – despite of the high sequence similarity – the *amlE* homologs from *Actinoplanes* might have evolved separately from those from *Streptomyces*.

Interestingly, *S. glaucescens* GLA.O annotates for a second *amlE*-homolog, which is part of the *gac* acarbose gene cluster. Here, comparable to the arrangement in *Actinoplanes* ssp., it occurs together with a LacI/GalR-like transcriptional regulator (GarC2, Rockser and Wehmeier, 2009). Presence of this gene arrangement in the *gac* operon of *S. glaucescens* is fascinating and raises the questions, if and how acarbose and sugar metabolism are interconnected with each other.

Both systems – the *aml* and the *acb* operon – are involved in the sugar metabolism, but with different areas of influence: Whereas AmlE is responsible for the intracellular assimilation of maltose, acarbose is involved in the extracellular metabolism acting as inhibitor of foreign α-glucosidases and carbophore (Wehmeier and Piepersberg, 2009). A regulatory balance of both systems might be vital, as maltose is substrate respectively precursor of both.

In SE50/110, both systems are separated spatially and regulatory. A direct involvement of AmlE in the acarbose biosynthesis or a direct regulatory effect by AmlR on the *acb* genes was not observed in this work. We therefore assume, that the *aml* and the *acb* operon have evolved separately in *Actinoplanes* sp. SE50/110. This hypothesis is supported by the fact, that the *aml* operon occurs in further species of the family Micromonosporaceae, but the *acb* gene cluster does not. Notwithstanding, in *S*. *glaucescens* GLA.O, both are included in one – the *gac* gene cluster – together with two regulatory genes: the LacI/GalR-type regulator mentioned before (GarC2) and a second regulator named GarC1 with homology to the acarbose regulator AcrC of SE50/110 described by Wolf et al. (2017a) and Rockser and Wehmeier (2009).

We suspect the evolution of the *acb* gene cluster as following: In SE50/110, the *acb* gene cluster has evolved earlier and was integrated into the global sugar metabolism. During this evolution, the regulator AcrC has switched its regulon from the genes *malEFG* to *acbD, acbE*, and *acbZ*, like assumed by Wolf et al. (2017a). In GLA.O, the *aml* operon and the acarbose biosynthesis gene cluster were incorporated at a later stage, which required simultaneous transfer of corresponding regulatory genes, namely the AmlR-homolog GarC2 and the AcrC-homolog GarC1.

In summary, metabolism of sugar and acarbose are intimately connected with each other as the synthesis of acarviosyl-metabolites requires sugar-precursors (Wendler et al., 2014), its production is strictly growth-associated (Wendler et al., 2014; Wolf et al., 2017a), and its product has been assumed to be involved in the acquisition of sugar molecules (Wehmeier and Piepersberg, 2004, 2009). A co-evolution of *amlE-amlR* gene arrangement along with the acarbose biosynthesis gene cluster is unlikely for *Actinoplanes* sp. SE50/110, but could have occurred for the species *S. glaucescens* GLA.O. This interconnection of sugar- and acarviosyl-metabolism needs further investigation.

### Transcriptome Analyses Reveal Lack of a Global Regulator of the Maltose/Maltodextrin System and Evince AmlR as Local Glucose-Dependent Transcriptional Repressor of the *aml* Operon

The expression of genes involved in the uptake and assimilation of maltose and maltodextrins has been described as sugar-dependent in various species, like *E. coli* (Boos and Shuman, 1998), *S. lividans* (Nguyen et al., 1997; Nguyen, 1999; Schlösser et al., 2001), *S. coelicolor* (van Wezel et al., 1997a, b) and *B. subtilis* (Schönert et al., 1998, 2006).

In *Actinoplanes* sp. SE50/110, a sugar-dependent expression of *mal* genes was assumed in the past, as first indications were provided by extensive proteome analysis (Wendler et al., 2015a). Indeed, a global transcriptional regulator of *mal* genes had not been found yet.

In order to gain further knowledge, we performed transcriptome analyses of triplicates of the wild type grown in maltose or glucose as main carbon source. We could confirm glucose-repression for *amlE* and *aglEFG*, which is in accordance to previous findings from Wendler et al. (2015a). It was striking, that no further gene of the maltose/maltodextrin metabolism was found to be differentially expressed. Therefore, the *mal* system of SE50/110 is not globally regulated in dependence of the sugars maltose or glucose.

By deletion of the PurR/LacI-type transcriptional regulator AmlR, a glucose-dependent repression of the *aml* operon was demonstrated. In Δ*amlR*, *amlE*, and *ACSP50_2473* are 23-fold respectively 25-fold higher transcribed compared to the wild type when cells were grown on glucose. A putative 12 nucleotide palindromic binding motif was identified in the promoter region of *amlE*, which meets the expectations of a PurR/LacI-type binding motif. The motif occurs only once in the genome of *Actinoplanes* sp. SE50/110.

We therefore suggest, that AmlR is the local repressor of the *aml* operon.

### Global Effects by Deletion of a Local Transcriptional Repressor and Putative Connection to the c-di-GMP Metabolism

The deletion mutant **Δ***amlR* displays severe growth inhibitions on glucose and a complex response on the level of the transcriptome. As AmlR operates as local transcriptional repressor and no further primary targets could be identified in this paper, we assume secondary effects by overexpression of *ACSP50_2473*.

ACSP50_2473 is a GGDEF-EAL tandem domain protein. *Actinoplanes* sp. SE50/110 harbors a total of 67 GGDEF or EAL domain proteins, of which 48 contain a GGDEF-EAL-tandem motif (data not shown). The presence of both domains with opposite enzymatic activities has been described as enzymatic conundrum (Ross et al., 1987; den Hengst et al., 2010; Hull et al., 2012; Römling et al., 2013; Mouri et al., 2017): On the one hand, one of the two domains might carry out novel functions in binding of substrates or in protein-protein interactions (Römling et al., 2013). On the other hand, both domains might be enzymatically active, but differentially regulated, which has been discussed controversially in the past (Römling et al., 2013). However, although the enzymatic function of ACSP50_2473 remains unclear, we think, that it is somehow involved in the c-di-GMP/pGpG metabolism.

Cyclic diguanylate is one of the most widespread second messengers in bacteria. It plays a vital role in signal transduction during processes like biofilm formation, motility, virulence, cell cycle and differentiation (Römling et al., 2013). C-di-GMP is conformational flexible and can bind as linear monomer, intercalated dimer or tetramer to various effector complexes (reviewed in Tschowri, 2016). Because of this flexibility, bioinformatic prediction of c-di-GMP binding sites is difficult: To date, 6 classes of c-di-GMP effectors have been described (reviewed in Tschowri, 2016). Also cross-system activation between nucleotide second messengers and hybrid c-AMP-GMP second messenger have been reported in the past (Fong and Yildiz, 2008; Pesavento and Hengge, 2009; Davies et al., 2012; Römling et al., 2013; Luo et al., 2015). Due to this, c-di-GMP can be involved at several regulatory levels, like allosteric regulation, transcriptional regulation and localized proteolysis and seems to respond to different environmental parameters (reviewed in Pesavento and Hengge, 2009). The sheer number of proteins with c-di-GMP responding or coordinating domain as well as the occurrence of a multiplicity of them in singular species, has raised the question, how signaling specificity can be achieved without detrimental cross-talk (Pesavento and Hengge, 2009; Seshasayee et al., 2010). It was assumed, that both global and local c-di-GMP concentrations determine the phenotypically output (reviewed in Pesavento and Hengge, 2009; Römling et al., 2013). According to this, computational analysis of 11,248 GGDEF and EAL-containing proteins from 867 prokaryotic genomes pointed out, that half of them contain a signal for cell-surface localization, by which their activity might be spatially separated (Seshasayee et al., 2010).

We found an N-terminal transmembrane domain in ACSP50_2473. Due to this and its organization within the *amlE-amlR* gene arrangement, a local effective range might be feasible, f. e. in giving feed-back to the transcriptional regulator AmlR. However, due to the strong growth inhibitions and complex transcriptomic response observed in Δ*amlR*, we assume a further involvement into the global “life-style switch” mechanism of c-di-GMP/pGpG.

We searched for indications for c-di-GMP/pGpG-mediated regulatory processes in Δ*amlR*. Indeed, transcriptome analyses displayed differential transcription of several genes of the secondary metabolite biosynthesis and of genes involved in sporulation, cell division, respiration, signal transduction, including 2 further GGDEF-EAL tandem proteins and 23 regulators of different regulator families. It is possible, that the transcription of these genes is modulated by c-di-GMP/pGpG-dependent effector complexes, which can cause global effects.

However, the nucleotide second messenger metabolism – in particular the one from c-di-GMP – is poorly understood (Pesavento and Hengge, 2009; Römling et al., 2013). Therefore, it is not possible to draw all consequences, that might be caused by overexpression of ACSP50_2473 on glucose. By pre-screening of the transcriptome of Δ*amlR*, it was possible to catch a glimpse of putative secondary effects caused by c-di-GMP/pGpG-effector complexes. We found a variety of putative target genes with wide-ranging functions concerning morphological and regulatory changes, which need further investigation.

It is highly interesting, that the *amlE-amlR* gene arrangement seems to be involved into global physiological processes. As maltose is one of the most important carbon sources of *Actinoplanes* sp. SE50/110, which is serving as preferred precursor of the acarviosyl-metabolite synthesis at the same time (Wendler et al., 2014), a coupling to other systems involved in growth and morphological changes seems to be reasonable. For a soil-inhabiting microorganism, which must adapt quickly to nutritional changes, it might be beneficial to decide flexibly between the utilization of maltose as energy source or as precursor of the acarbose biosynthesis, which might secure further C-sources via the carbophore-mechanism or by inhibition of microbial competitors.

## Data Availability Statement

The datasets generated for this study can be found in the ArrayExpress database, https://www.ebi.ac.uk/arrayexpress/experiments/E-MTAB-8404/ (accession no. E-MTAB-8404).

## Author Contributions

LS designed, planned, and interpreted the experimental work, drafted the manuscript, and performed the *in silico* studies, growth experiments of Δ*amlE*, enzymatic assays, and transcriptome analyses. LS and JD constructed the deletion mutants Δ*amlE* and Δ*amlR* by CRISPR/Cas9. SD supported the laboratory work concerning the growth experiments of Δ*amlR* and substrate analytics. TB sequenced the RNA-library. DB performed the data processing and mapping. JK, SS-B, and MP assisted in interpreting the data and revised the manuscript. JK and AP coordinated the study.

## Conflict of Interest

The authors declare that the research was conducted in the absence of any commercial or financial relationships that could be construed as a potential conflict of interest.

## Footnotes

1 https://blast.ncbi.nlm.nih.gov/Blast.cgi

2 https://www.ncbi.nlm.nih.gov/cdd

3 http://www.cbs.dtu.dk/services/TMHMM/

4 http://dendroscope.org/

5 https://edgar.computational.bio.uni-giessen.de/cgi-bin/edgar_login.cgi

6 http://meme-suite.org/tools/meme

7 http://meme-suite.org/tools/fimo
