# Supplementary material for: Essentiality of the Maltase AmlE in Maltose Utilization and Its Transcriptional Regulation by the Repressor AmlR in the Acarbose-Producing Bacterium Actinoplanes sp. SE50/110
Source: Front Microbiol. 2019 Oct 29;10:2448. doi: 10.3389/fmicb.2019.02448 (PMC6828939; doi:10.3389/fmicb.2019.02448)
Supplement: Supplementary file 1 [file Data_Sheet_1.docx]

Supplementary Material file 1

**Supplementary material file 1** includes all supplementary figures and tables.

Further data provided as xlsx-file (Supplementary material file 2): List of significant differentially transcribed genes in the regulator mutant ∆*amlR* compared to the wild type when grown on glucose minimal medium.

## Supplementary Figures


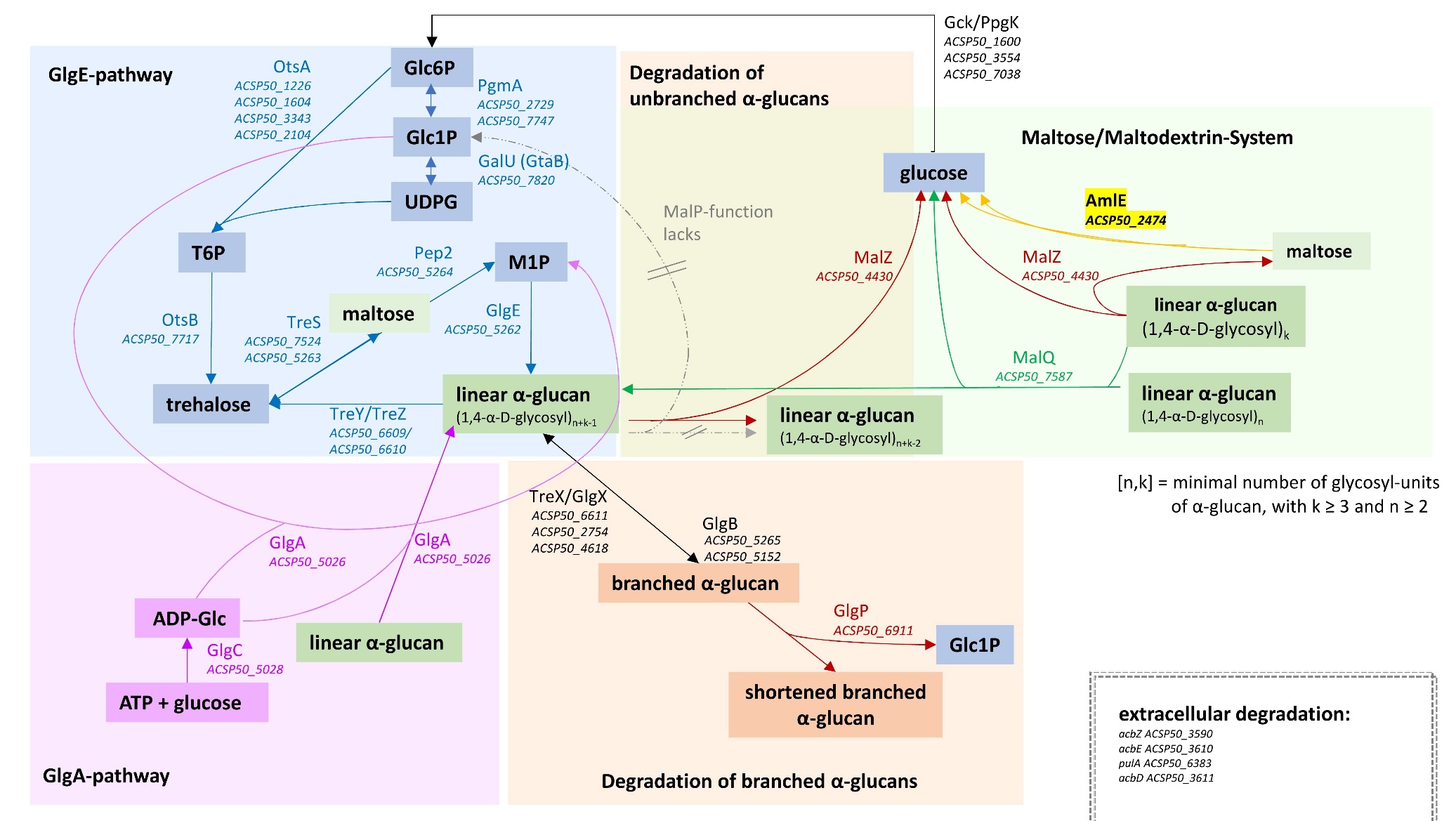


**Supplementary figure 1:** Model of the maltose/maltodextrin/glycogen metabolism in *Actinoplanes* sp. SE50/110 according to homology comparison to other microorganisms (see description below) and including the improved functional annotation (Wolf, T. et al. 2017) as well as studies from *Actinoplanes* (Lee, Jin‑Sook et al. 2008). Further literature is referenced in the description below and in supplementary table 3.

**Comment on the model in supplementary figure 1:**

Linear α-glucans can be built up by three different pathways: the **GlgE-**, the **GlgA-** and the **maltose/maltodextrin pathway**.

**MalQ** recognizes small maltodextrins with minimal size of three glycosyl-units, removes glucose from the reducing end and transfers the maltodextrinyl-residue to the non-reducing end of other maltodextrins (minimal size: two glycosyl-residues) (Boos, Winfried und Shuman, Howard 1998). By this, glucose and a chain-extended maltodextrin of a minimal length of four glycosyl-units is released in *E. coli.* The glucose is removed from the reaction equivalent by phosphorylation and passed on to the glycolysis (Boos, Winfried und Shuman, Howard 1998). Linear glucans of a minimal size of four glycosyl-residues are degraded by **MalP**-phosphorylase in *E. coli* (Boos, Winfried und Shuman, Howard 1998; Park, J.‑T. et al. 2011) and *C. glutamicum* (Seibold, Gerd M. et al. 2009). The released glc-1P is directed to the glycolysis for growth and energy production but also fuels the formation of ADP-glucose and subsequently the formation of glycogen via the **GlgA**-pathway.

The glucosidase **MalZ** is proposed as hydrolase, which has shown to release glucose from the reducing end with maltotriose as smallest substrate in *E. coli* (Tapio et al. 1991; Boos, Winfried und Shuman, Howard 1998). **MalZ** has not been identified in *C. glutamicum* (Seibold, Gerd M. et al. 2009). The periplasmic hydrolase **MalS** only occurs in the Gram-negative organism *E. coli* and is not shown here.

In the **GlgA**-pathway, linear 1,4-α-glucans are built up by the activity of the glycogen synthase **GlgA** from energy-rich ADP-glucose, which is supplied by **GlgC** (Wilson et al. 2010; Seibold, Gerd et al. 2007).

In the recently discovered **GlgE** pathway, maltose-phosphate is used as substrate instead, which is derived from trehalose by isomerization (**TreS**) and phosphorylation (**Pep2/MAK**) (Kalscheuer et al. 2010). In both pathways, **GlgB** introduces branches into the linear glucan leading to the storage substance glycogen (Chandra et al. 2011). Within the **GlgE-**pathway, the debranching enzyme **TreX**, the maltooligosyl-trehalose-synthase **TreY** and the hydrolase **TreZ** form a cycle by splitting off trehalose from glycogen (Chandra et al. 2011). According to Chandra *et al.* (2011), the **GlgE**-pathway was found in 14% of all sequenced genomes from diverse bacteria. It occurs also in closely relatives of *Actinoplanes*sp. SE50/110, like *S. coelicolor* (Chandra et al. 2011; Schneider et al. 2000) and *C. glutamicum* (Woo et al. 2010). This pathway was well studied in the actinomycete *S. venezuelae*, which possesses **GlgE** but no **GlgA** pathway (Miah et al. 2016).

Recent studies from *Mycobacterium tuberculosis* have pointed out the direct linkage between the **GlgA**- and the **GlgE**-pathway, by postulating, that α-glucans in mycobacteria are exclusively assembled by α-maltose-1-phosphate moieties, which is deployed by both **TreS/Pep2** as well as **GlgA**. Therefore, **GlgA** is able of directly producing α-maltose-1-phosphate from ADP-glucose and glucose-1-phosphate (Koliwer-Brandl et al. 2016). This is in accordance with previous findings, that prokaryotic **GlgA** does not require primer molecules for the glycogen elongation process (Wilson et al. 2010).


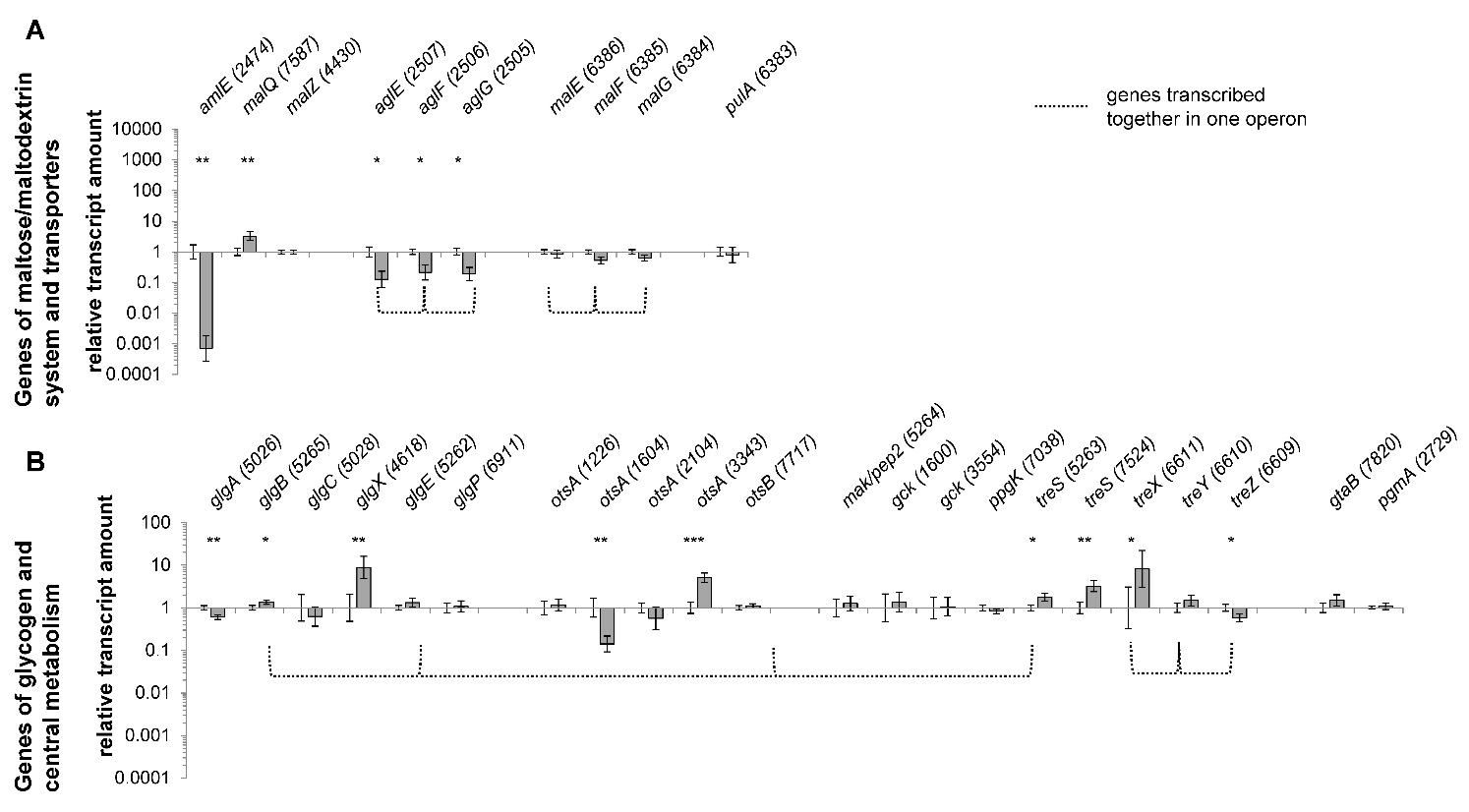


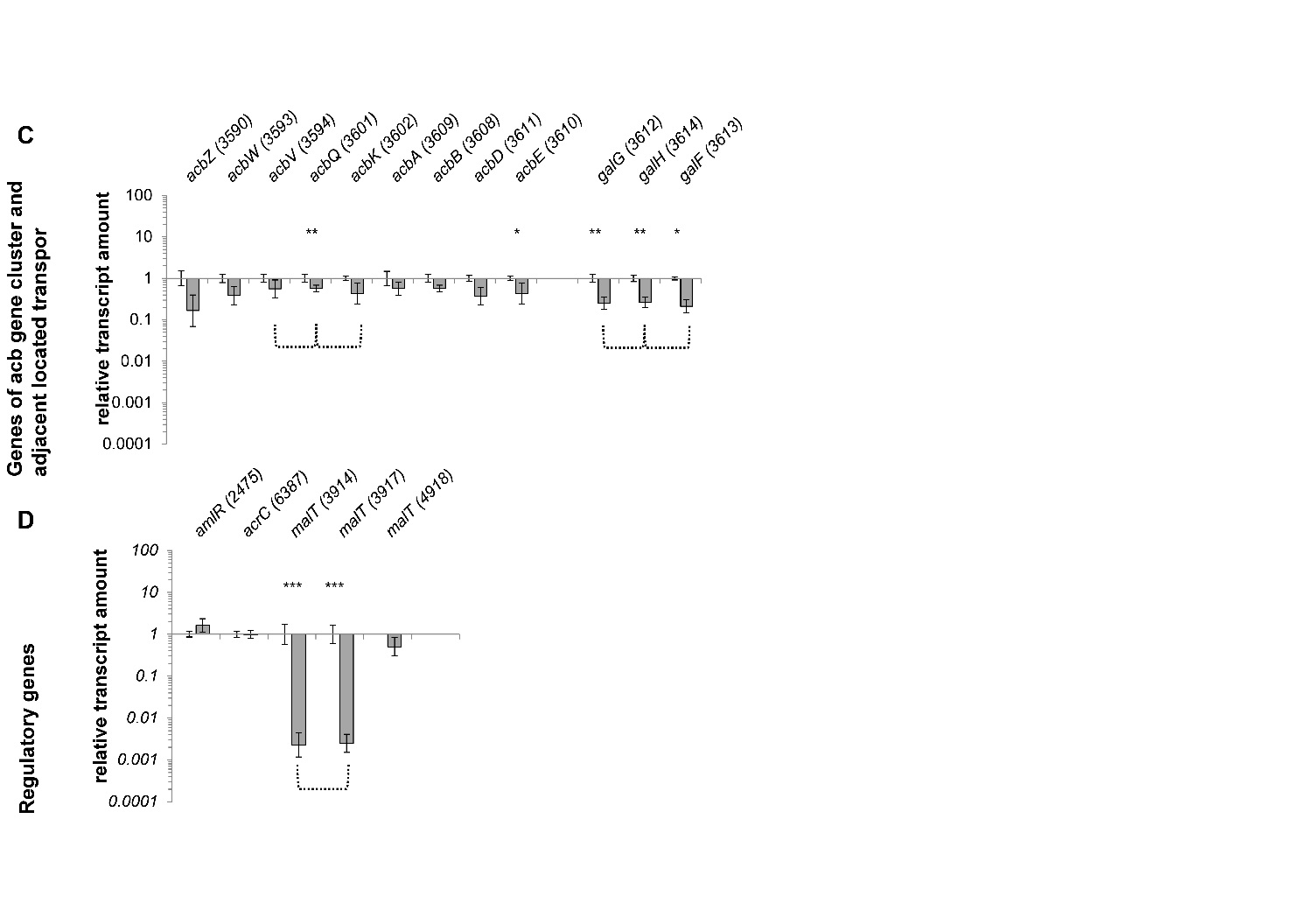


**Supplementary figure 2:** Relative transcription of genes of the glycogen/maltose/maltodextrin metabolism in glucose- compared to a maltose-grown culture of *Actinoplanes*sp. SE50/110 (relative transcription of the genes on maltose set to 1). Significance of differential transcription was calculated by a two-sided t-test indicating: ***** p-value < α = 5 %, ****** p-value < α = 1 %, ******* p-value < α = 0.1 %. Part A corresponds to **figure 4** of the main text.


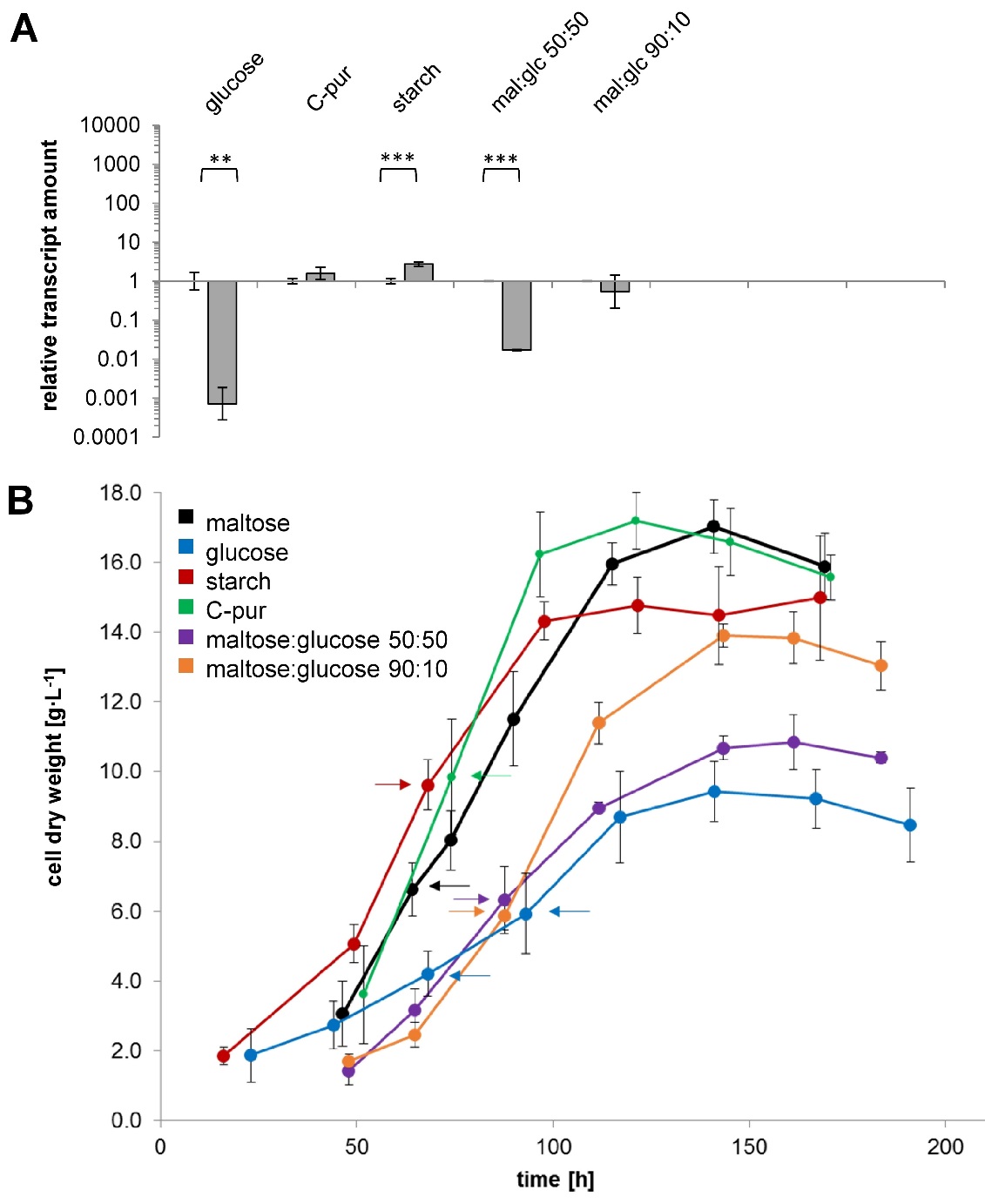


**Supplementary figure 3:** Relative transcript amounts of the gene *amlE* in the wild type of *Actinoplanes*sp. SE50/110 (**A**), when grown on the carbon sources glucose, C-Pur, starch and mixtures of maltose and glucose in the ratio 50:50 and 90:10 compared to maltose-grown culture. Arrows in the growth curves (**B**) indicate sampling times for transcriptome analyses.

Whereas on glucose, *amlE* transcription is round about 1,385-fold reduced compared to the maltose-grown culture, expression is similar on C-Pur and slightly enhanced on starch (2.75-fold). In a 50:50 mixture of glucose and maltose, repression in presence of glucose becomes apparent, too, as here, the expression of the gene *amlE* was 580-fold reduced. In a 90:10 mixture of maltose and glucose, glucose is quickly depleted at the beginning growth phase according to our substrate analytics (data not shown). Therefore, glucose has already been exhausted at RNA sampling time, which is why the relative transcript amount of *amlE* is similar compared to the maltose-grown culture.

Significance of differential transcription was calculated by a two-sided t-test. Asterisks indicate the significance level (* p‑value < α = 5 %, ** p‑value < α = 1 %, *** p‑value < α = 0.1 % (p-values: glc: 0.001533, C‑pur: 0.1439, starch: 0.0004504, mal:glc 50:50: 4.204e-05, mal:glc 90:10: 0.6907).


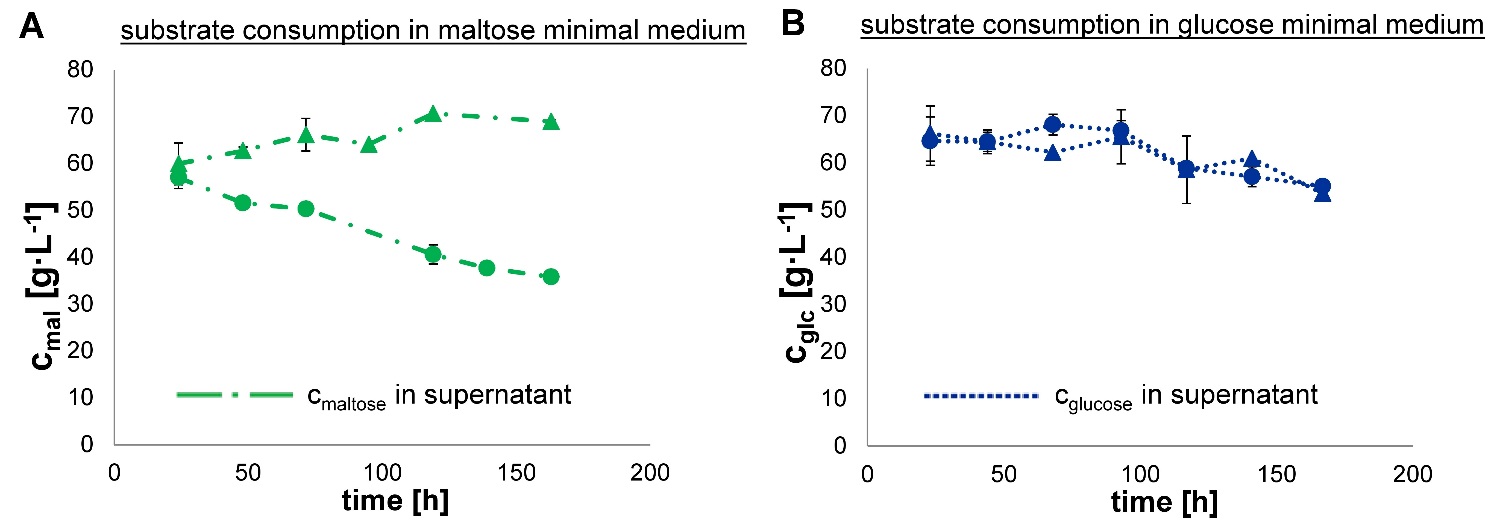


**Supplementary figure 4:** Substrate analytics of the wild type (●) and the deletion mutant ∆*amlE* (▲) of *Actinoplanes*sp. SE50/110 in minimal medium supplemented with maltose (**A**) or glucose (**B**) as carbon source.


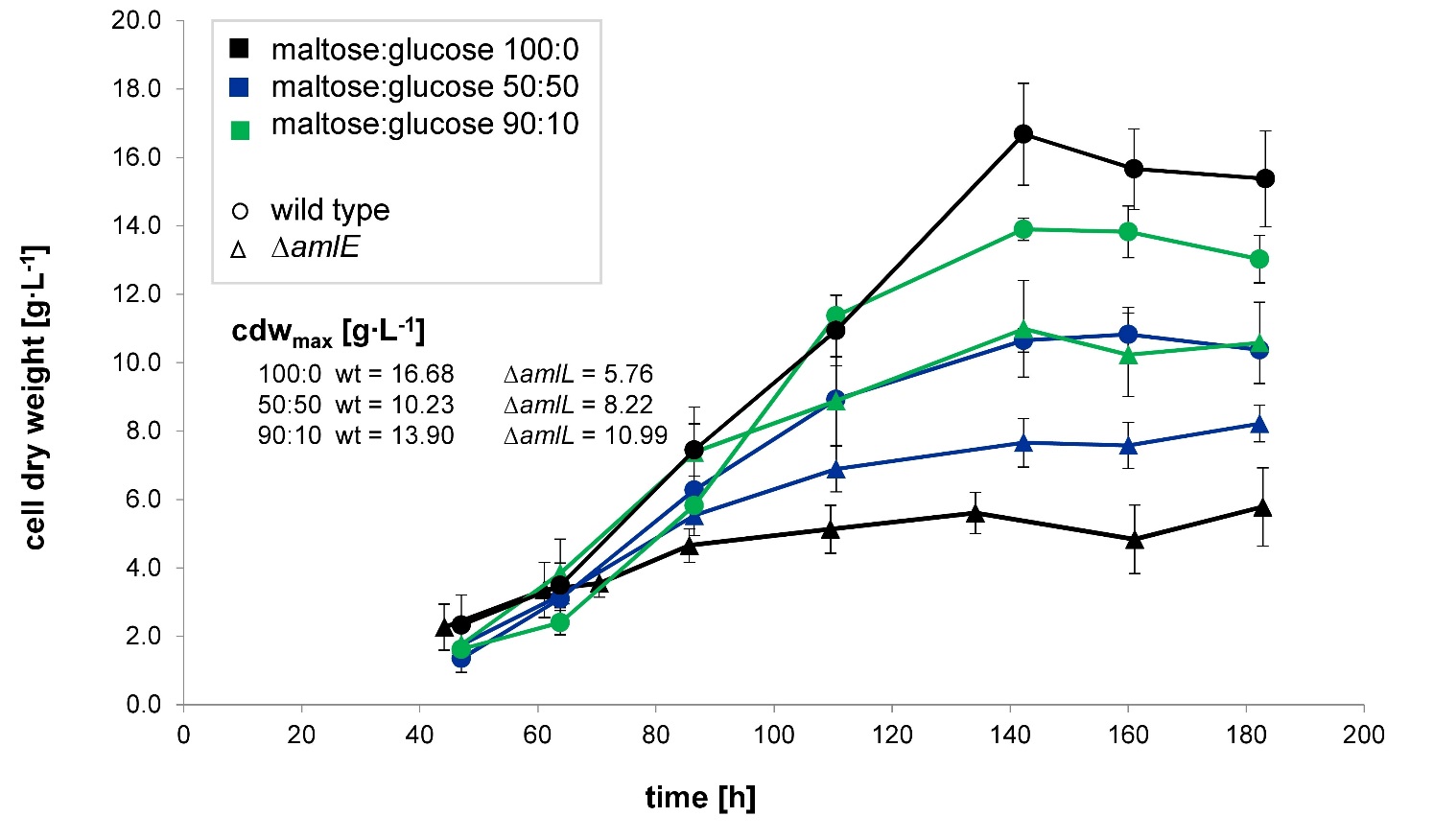


Supplementary figure 5: Growth of the wild type (●) and the deletion mutant ∆*amlE* (▲) of *Actinoplanes*sp. SE50/110 in minimal medium supplemented with different mixtures of glucose and maltose: maltose:glucose 50:50 (dark-blue, n_∆_*_amlE_* = 4, n_wt_ = 2), maltose:glucose 90:10 (green, n_∆_*_amlE_*= 5, n_wt_ = 4). The deletion mutant ∆*amlE* displays reduced growth compared to the wild type These differences are significant according to a two-sided t-test of the final cell dry weights (50:50: p-value = 0.01252, 90:10: p‑value = 0.0002094).


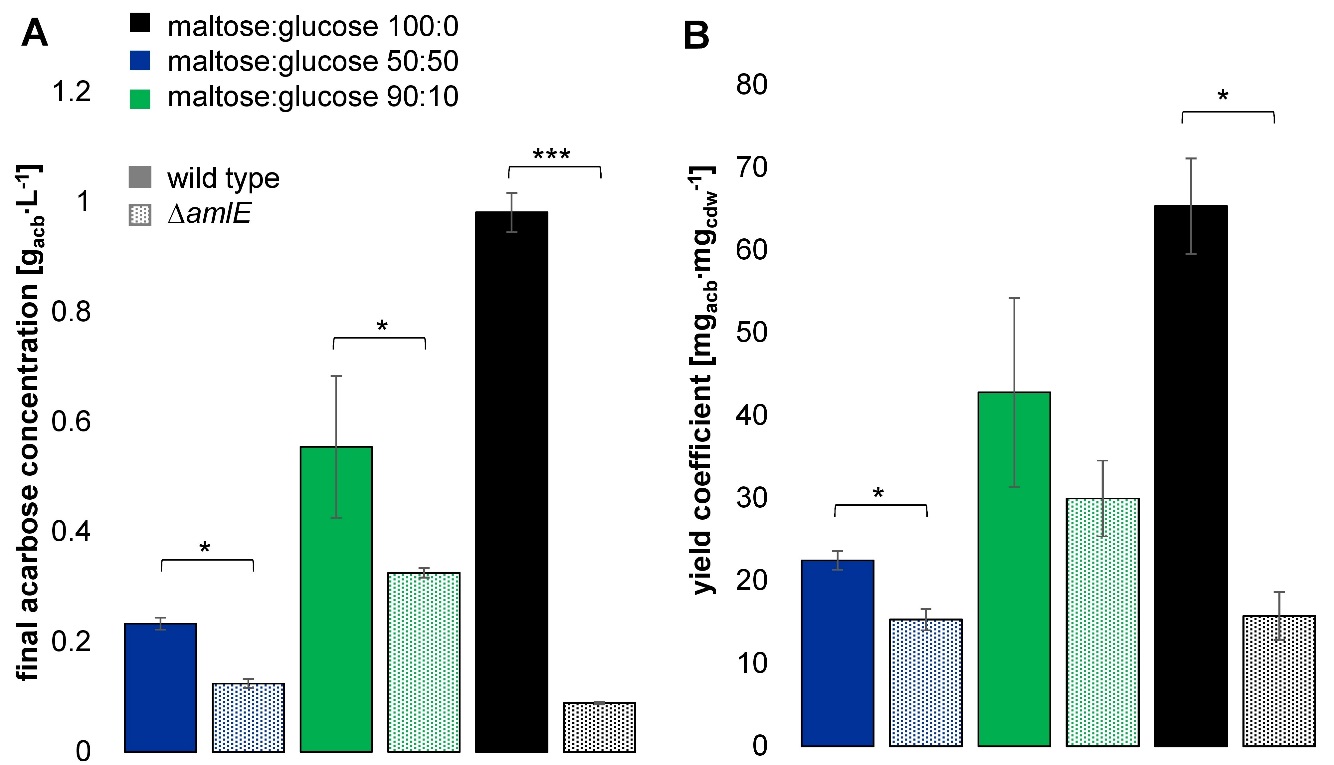


**Supplementary figure 6:** Final acarbose concentrations (**A**) and biomass-related product yield coefficients (**B**) of the wild type (filled columns) and the deletion mutant ∆*amlE* (checkered columns) of a shake flask cultivation in maltose minimal medium (black) and in minimal medium supplemented with different mixture of glucose and maltose (maltose:glucose 50:50 (dark-blue), maltose:glucose 90:10 (green)). Significant differences are denoted by asterisks (differences in final acarbose concentrations between wild type and ∆*amlE:* p-values (color-coded): 0.13356262, 0.01112, 7.409e-05, differences in yield coefficient between wild type and ∆*amlE:* p-values (color-coded): 0.13356262, 0.01112, 7.409e-05). The corresponding growth curves are shown in **figure 5A** (maltose minimal medium) respectively **supplementary figure 5** (mixtures of glucose and maltose).


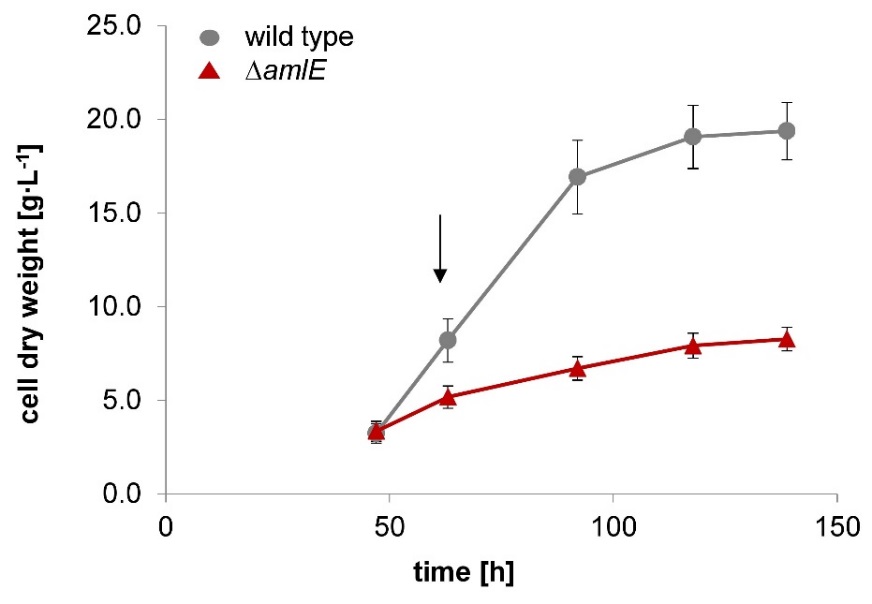


Supplementary figure 7: Growth of *Actinoplanes*sp. SE50/110 wild tyoe (●) and the deletion mutant ∆*amlE* (▲) in minimal medium complemented with C-Pur as carbon-source. Arrow indicates sampling times for performance of the activity assays.


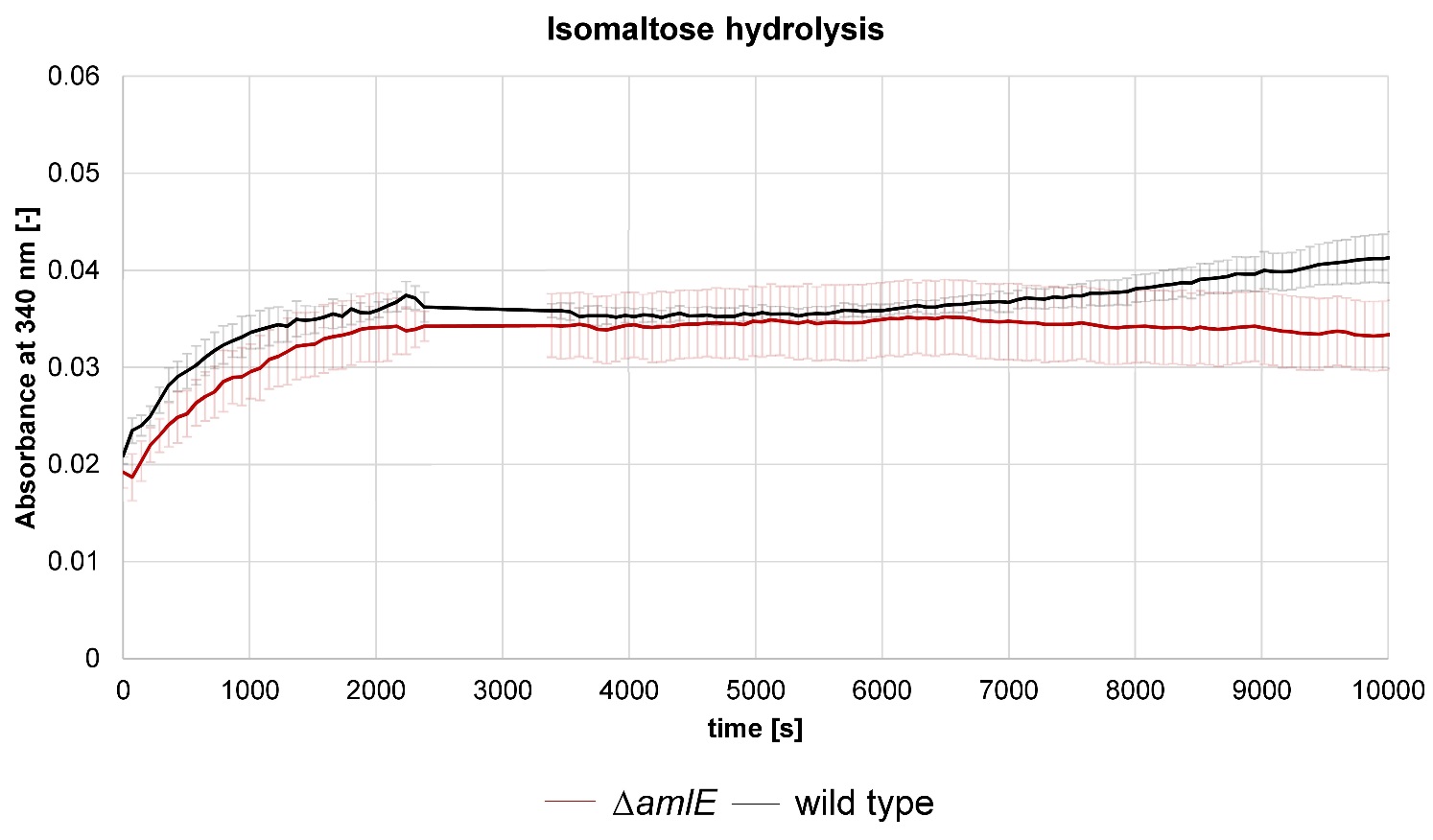


Supplementary figure 8: NADPH-release from enzymatic assays testing for the hydrolytic activity of protein raw extract from the wild type and the deletion mutant ∆*amlE* of *Actinoplanes*sp. SE50/110 concerning isomaltose, an α-1,6-linked disaccharide. Shown are the means and standard deviations of triplicates.


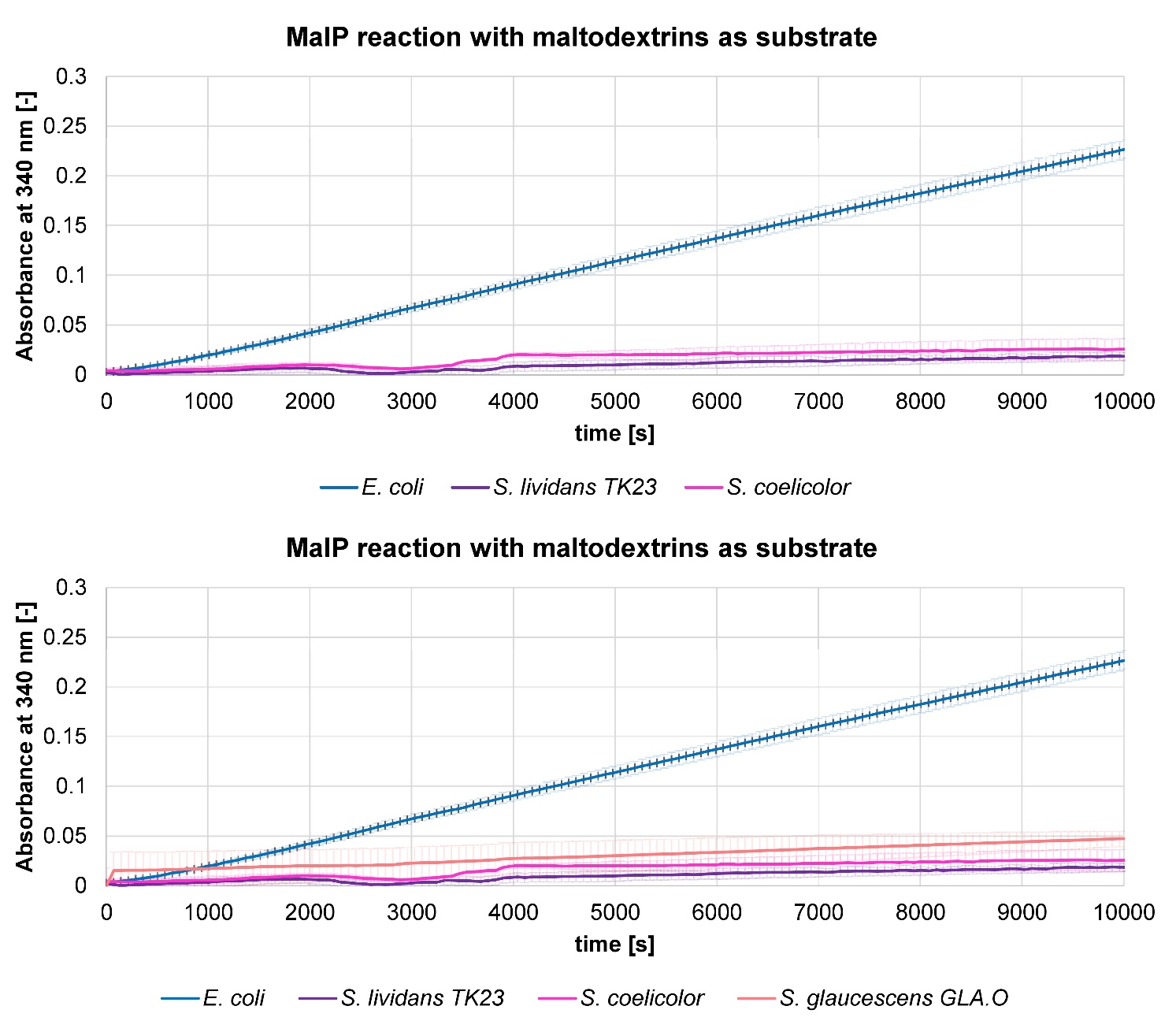


Supplementary figure 9: NADPH-release from a MalP enzyme assay measured by absorbance at 340 nm of protein raw extracts from *E. coli* DH5α, *S. lividans*TK23, *S. coelicolor* A3(2) and *S. glaucescens*GLA.O. Shown are the means and standard deviations of triplicates.


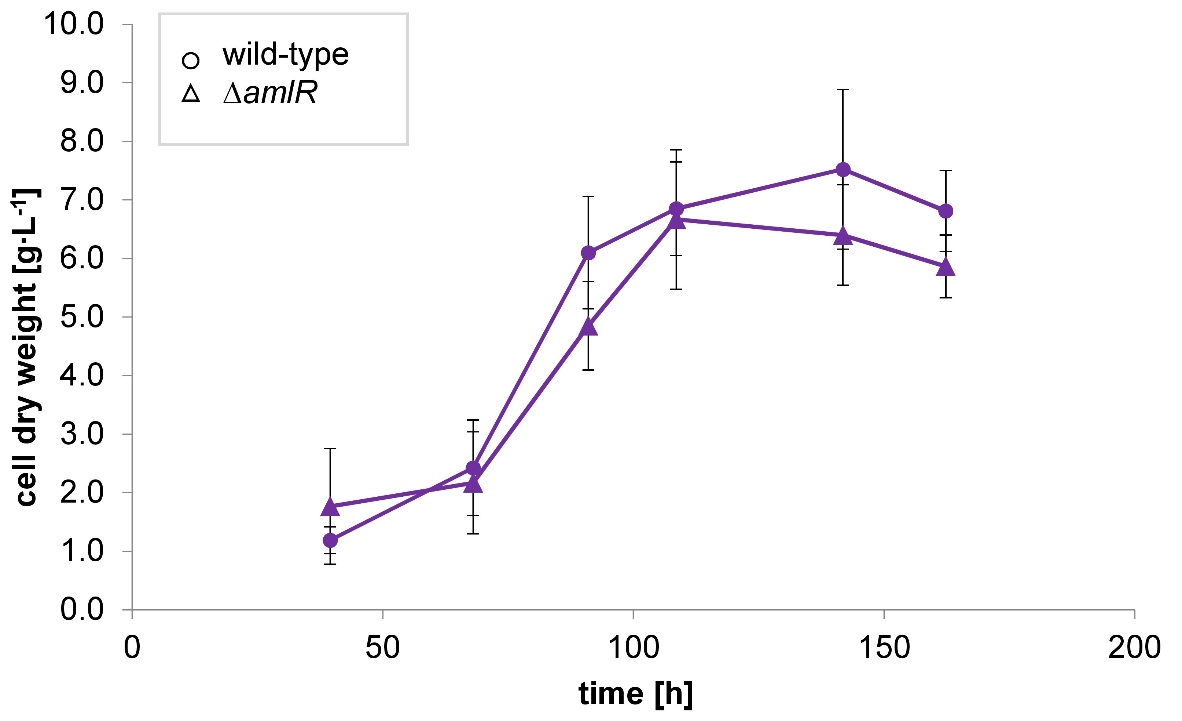


**Supplementary figure 10:** Growth of the wild type (●) and the deletion mutant ∆*amlR* (▲) of *Actinoplanes*sp. SE50/110 in a mixture of maltose and glucose minimal medium in the ratio of 80:20.


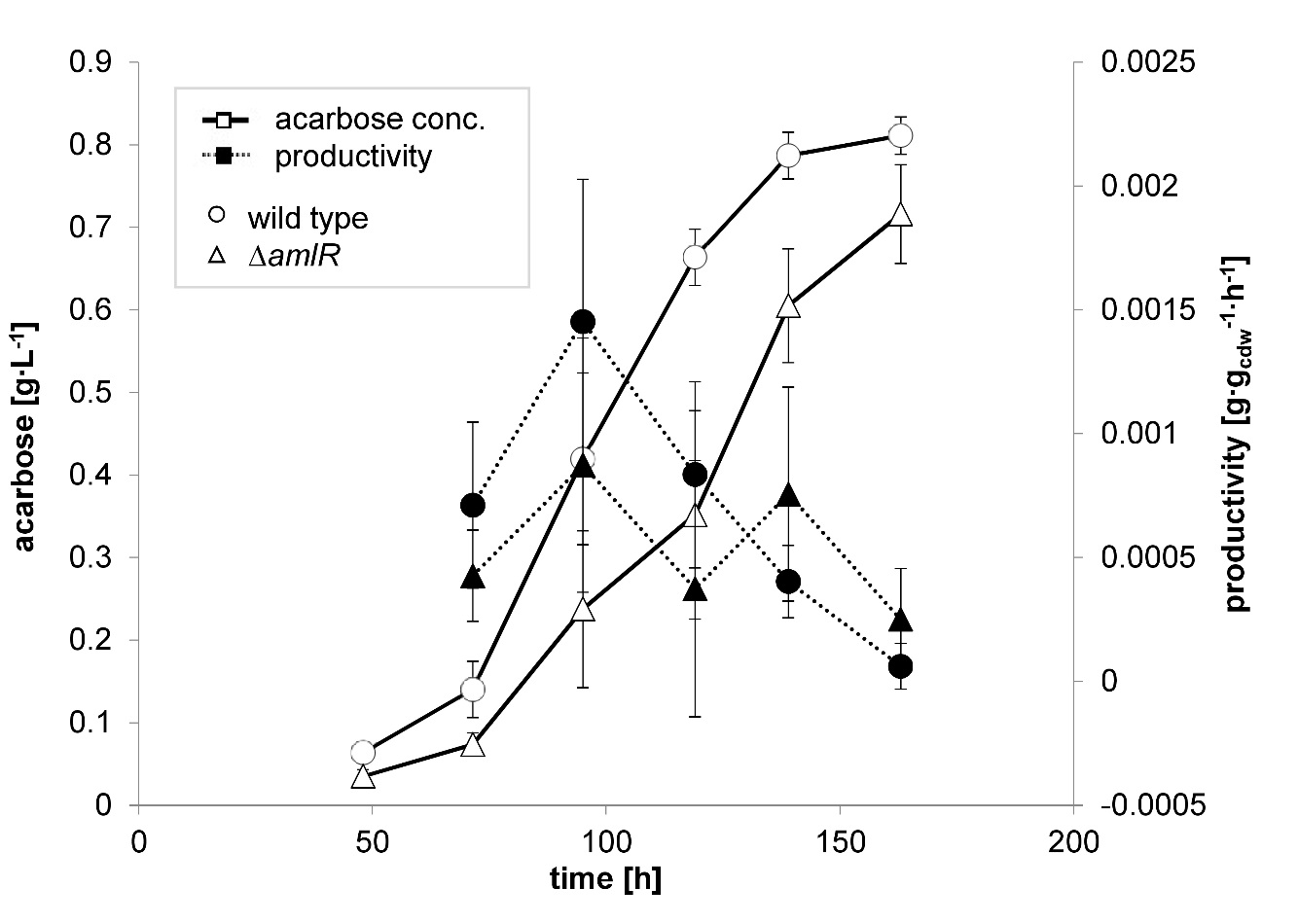


**Supplementary figure 11:** Acarbose concentration measured from the supernatant of a maltose-grown culture and biomass-related productivity of the wild type (●) and the deletion mutant ∆*amlR* (▲) of *Actinoplanes*sp. SE50/110.

**
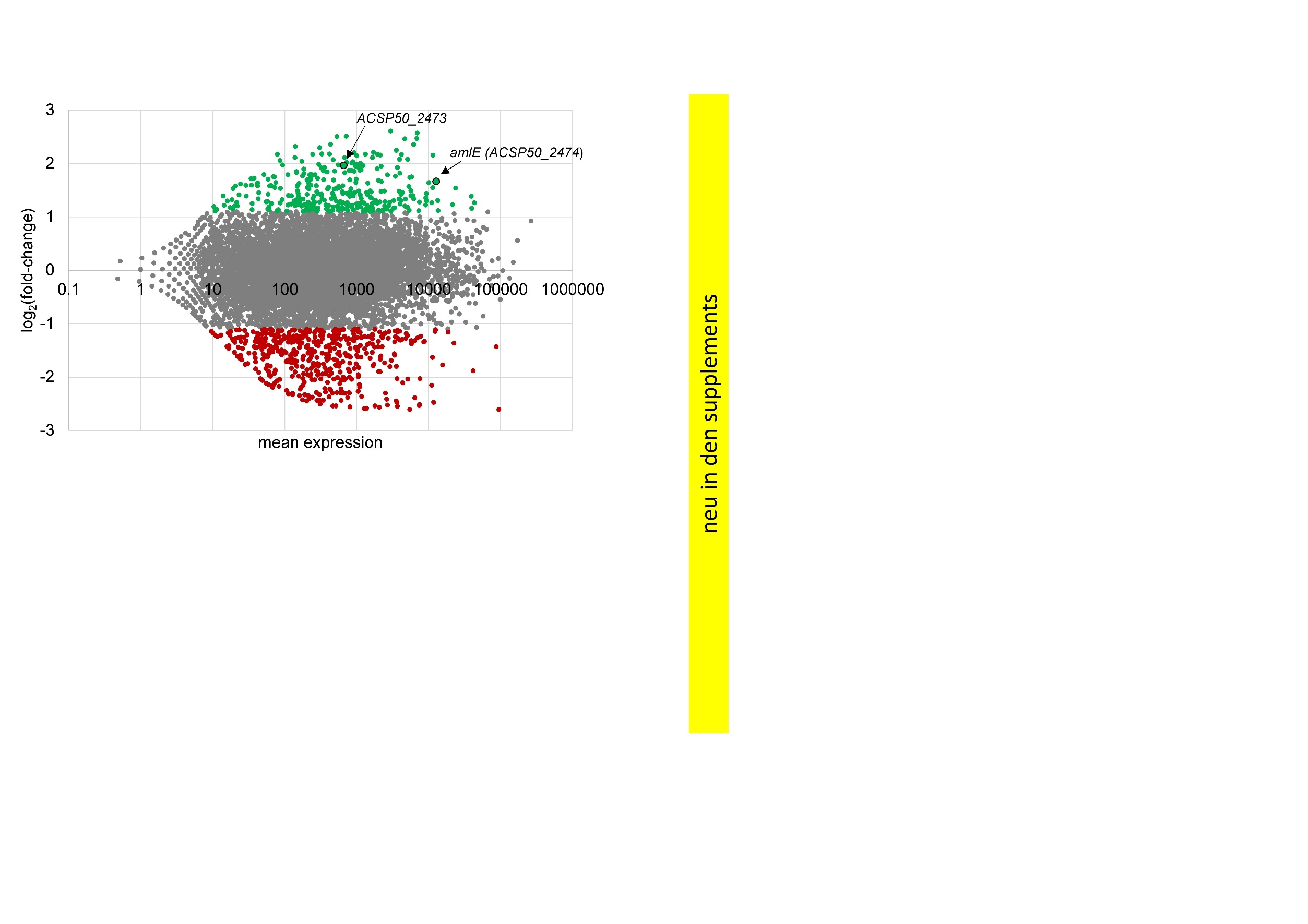
**

Supplementary figure 12: Ratio/intensity plot of differentially transcribed genes in ∆*amlR* compared to the wild type in glucose minimal medium by Deseq2-analyses (Love et al., 2017). The ratio (log_2_(fold-change)) is plotted against the mean average intensity. Green and red dots represent genes with significantly different transcript levels in the Δ*amlR* strain.


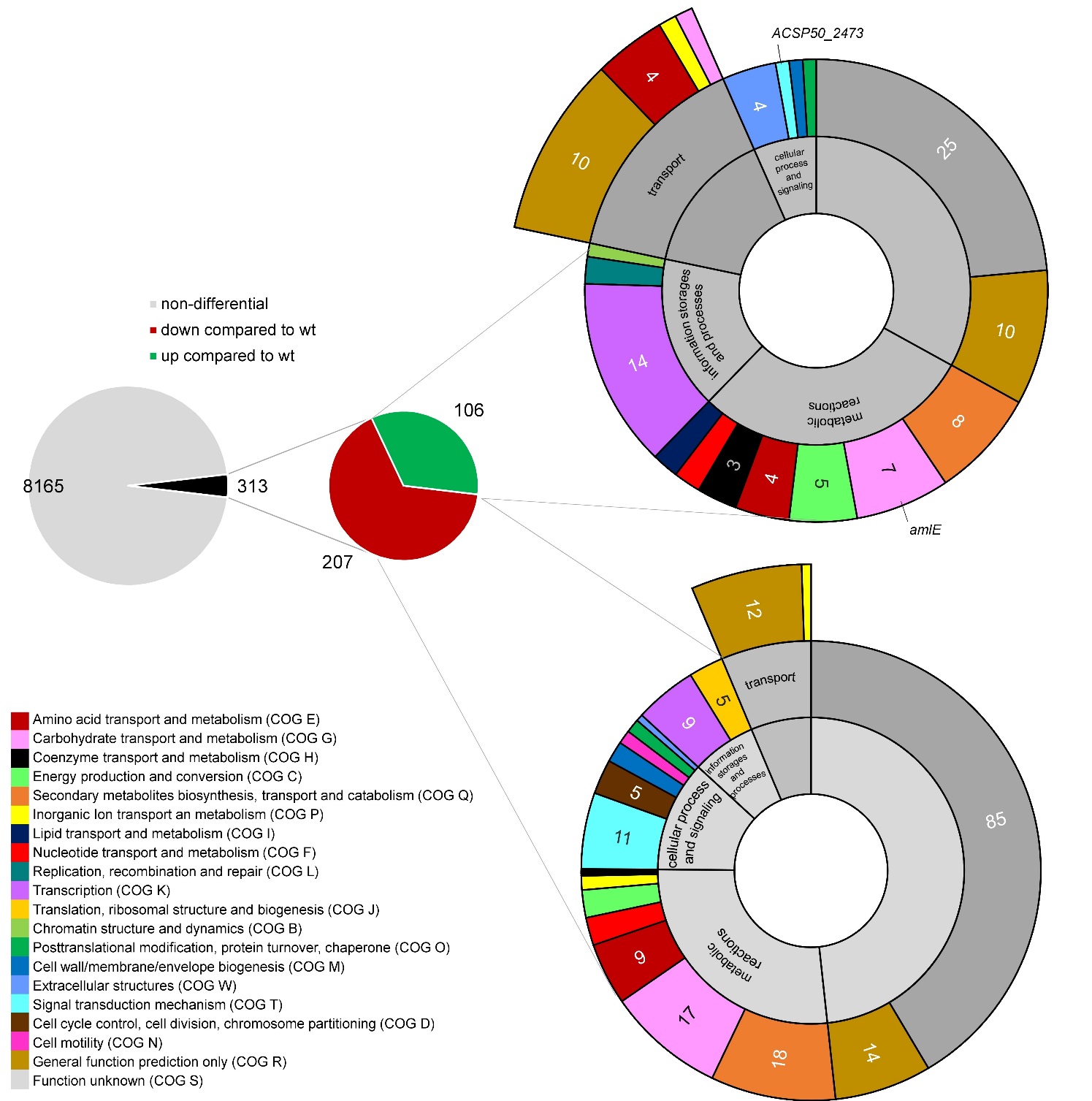


**Supplementary figure 13:** COG-classes of genes with significant increased respectively decreased transcript amounts in the regulator mutant ∆*amlR* on glucose minimal medium according to Deseq2-analyses (Love et al. 2017).


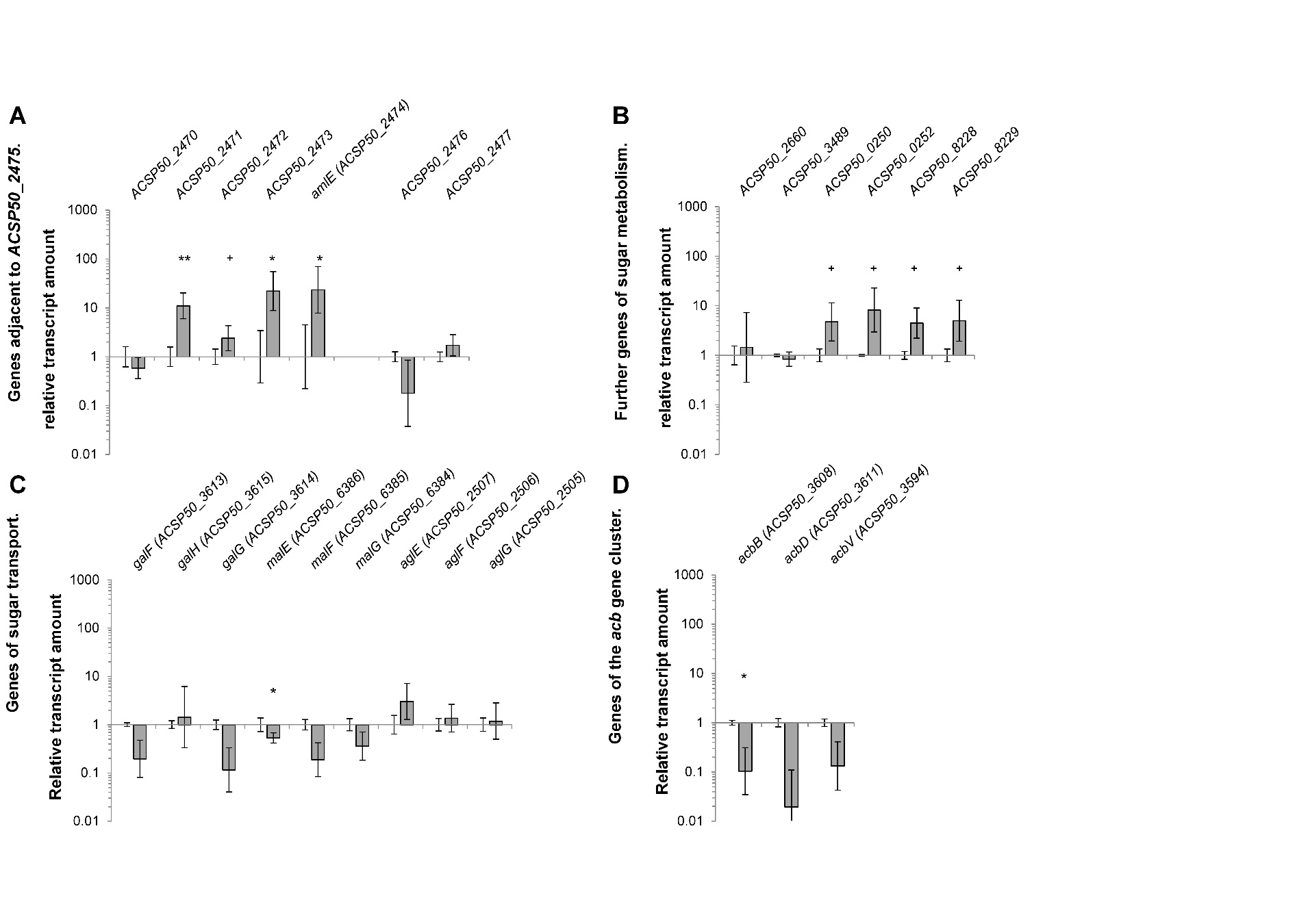


Supplementary figure 14: Relative transcription measured by RT-qPCR in the deletion mutant ∆*amlR* compared to the wild type of *Actin*oplanes sp. SE50/110, when cells are grown on glucose. Significance of differential transcription was calculated by a two-sided t-test indicating: + p-value < 10 %, * p-value < α = 5 %, ** p-value < α = 1 %, *** p-value < α = 0.1 %. The data are in accordance with the results from RNAseq (supplementary file 2).

## Supplementary Tables

**Supplementary Table 1.** Oligonucleotides for spacer annealing and primer sequences for Gibson Assembly*.*

| **primer name** | **primer sequence (5’-3’)** | **product (bp)** |
| --- | --- | --- |
| amlE_spacer_for | acgcGTCGAGCCAGAACCGCAGGA | **-** |
| amlE_spacer_rev | aaacTCCTGCGGTTCTGGCTCGAC | **-** |
| amlR_spacer_for | acgcGTAGGCCAGGACGTCGGAAA | **-** |
| amlR_spacer_rev | aaac TTTCCGACGTCCTGGCCTAC | **-** |
| amlE_fl1_GAF | tcggttgccgccgggcgttttttatAGGGCGAACATCAGTCCGAG | 1050 |
| amlE_fl1_GAR | gaaagaaagcagccgtcaggCGTGTGGTGGTCCGTCTAAG |  |
| amlE_fl2_GAF | cttagacggaccaccacacgCCTGACGGCTGCTTTCTTTC | 1029 |
| amlE_fl2_GAR | gcggcctttttacggttcctggcctAACAATCCGCAGCGGATCTC |  |
| amlR_fl1_GAF | ggttgccgccgggcgttttttatctagGTTCCACGCGGTGCCCAGGTAGC | 1047 |
| amlR_fl1_GAR | TCCGCGCCTCATCGGTCACACGACGGCTTGCAAGAACTTG |  |
| amlR_fl2_GAF | GTTCTTGCAAGCCGTCGTGTGACCGATGAGGCGCGGATGC | 1034 |
| amlR_fl2_GAR | tttacggttcctggcctGTTCGGCCGCGACCGGGCCCGCGGCTTTCG |  |

**Supplementary Table 2.** Primers used in qRT-PCR.

| **genetic locus** | **fwd-primer (5’-3’)** | **rev-primer (5’-3’)** | **fragment size (bp)** |
| --- | --- | --- | --- |
| *ACSP50_0250* | GTACGCGATCGTGGCCTCCG | ACTCGGACGGCAGCAGCAG | 184 |
| *ACSP50_0252* | ATGACGACGGTCGCAGTAAC | CACGTTGGTGTTCACATAGG | 303 |
| *otsA (ACSP50_1226)* | CTTCGTCGTCGTCGCCAACC | ACCGGGTGGATGTGGATGCC | 204 |
| *gck* (*ACSP50_1600*) | GTCGAGGTGGCCGCCGAG | CCACCCCGAACCGGAACTC | 225 |
| *otsA (ACSP50_1604)* | CGCTGAGCGATGACGAACTG | ACGAGTTGCAGGTGGTAGTC | 211 |
| *otsA (ACSP50_2104)* | TCCGGTTGAGCGAACAGGAC | GCACCAGCTGCAACTGGTAG | 216 |
| *ACSP50_2470* | TGGTCGCGCTGGGCCTGCTG | CTCGGCGATCACCCGCAG | 265 |
| *ACSP50_2471* | AGGCCATCCGCATCGCCTAC | TCGAGCACGAAGGCGACCTG | 240 |
| *ACSP50_2472* | GCACGCTGCTGCGGCTCATC | CGCCGGAGATCTGCCAGGTG | 264 |
| *ACSP50_2473* | CTCGGCCTGCGTGCTGAC | CCGGCATCGACCAGAGGAAG | 251 |
| *amlE (ACSP50_2474)* | AGCACGAGTGGTTCCAGAAG | GGGTGCTCCCAGTTGAAGTC | 214 |
| *amlR (ACSP50_2475)* | GTCGTTCGCGTTCAACAATC | GGTGAGCAGCAGGGTGTAAC | 247 |
| *csbD (ACSP50_2476)* | TCAAGGAGGGCGTCGGCAAG | GGACTTGAAGACGTCCTTGA | 119 |
| *ACSP50_2477* | CGTGGGTGGCGGTCACG | AGGGTGGGCGAGATCAGC | 234 |
| *ACSP50_2660* | GTGCGTGGCCTCAAGTTCAG | TGCGGTGCAGGTTGATGGTG | 175 |
| *aglE (ACSP50_2507)* | GTTCGGTGTTCAGCCAGTTC | CTTGGTCCAGAACTTGTC | 308 |
| *aglF (ACSP50_2506)* | GGCAAGCGCTCCGACAAC | ACGACTCGAACCGCGACTTG | 286 |
| *aglG (ACSP50_2505)* | ATCGTGATCGCGGTGCTCTG | CCACGAACAGGATGTTCTTG | 295 |
| *pgmA (ACSP50_2729)* | CTGTTCATGGGCCGCGACAC | CCCGATTCTTCCTGTTGTAG | 166 |
| *otsA (ACSP50_3343)* | TGCGCCAGCAGGGTGGTG | GCCTGGGCGAAGCGACGG | 244 |
| *ACSP50_3489* | GCTGTGGTGGCTCACCATTG | CCCGTACTCGTAGATGTTCC | 238 |
| *gck* (*ACSP50_3554*) | CGAGCGGTACGCGTCCAC | CACCCGGCGGGCGTAGATCG | 277 |
| *acbZ (ACSP50_3590)* | CGGCAATTCGCTGTTCAGTG | TGTGCTTGACGGTGTCCATC | 167 |
| *acbW* (*ACSP50_3593*) | GGTGTACGACCGGAACATGC | GTTCGGCGTGGATGTGGTTG | 224 |
| *acbV (ACSP50_3594)* | GCTTCCACGGCAAGACGATG | GCGCTCACGTTGGGTTTCTC | 196 |
| *acbQ (ACSP50_3601)* | TGCTGGCGCAGATCTACTCC | AGCCGCAGATACATCGGGTC | 211 |
| *acbK* (*ACSP50_3602*) | CGAGGTCTACGCCTTCAACG | AGAGGAAGCCGGACACGAAC | 248 |

Supplementary Table 2 continued

| *acbB (ACSP50_3608)* | CCCGCTGCTCGAACAACTAC | CCGCCGATGTGATAGACCTC | 205 |
| --- | --- | --- | --- |
| *acbA (ACSP50_3609)* | TCATGCTCGGCGACAACCTG | GACCGGTTTCTCCTCGATGG | 173 |
| *acbE (ACSP50_3610)* | GCGCGGCATGAAGATCTACC | CGGACGGCTTCTCGAAGAAC | 218 |
| *acbD (ACSP50_3611)* | ACGCCAACTACTGGATGGAC | TCGAGCGGTTGGTGTAGAAG | 231 |
| *galF (ACSP50_3613)* | TACTGCTCGACCTCGCCTTC | TCGATCACCACCGCGAACAG | 283 |
| *galG (ACSP50_3614)* | AACGTACCGGATCGCAGCTC | GGAATGTTGAACCGGAAACC | 295 |
| *galH (ACSP50_3615)* | GGCGACCCATGACACGTTTC | TGTTGTGGTCCGTGGTGTTG | 267 |
| *malT (ACSP50_3914)* | CTCTTCGCCGAACTGTTG | GTGCCTTCCACGATCAAC | 206 |
| *malT (ACSP50_3917)* | CAGGCGGTGACATTGATCTG | AGAAGGTGTACGGGTCGTTG | 139 |
| *malT (ACSP50_4918)* | CGTGATCAGCGGCCCGATGG | AGGTGCAGATCGTCGATGAG | 261 |
| *malZ (ACSP50_4430)* | CGTTTCGCGCCGTGGCTG | GATTGAACACGCCGTCGAAC | 208 |
| *glgX (ACSP50_4618)* | AAGAGATCTGGCCGGGCAAC | GTCGATGTCCGAGTCGATGG | 311 |
| *glgA (ACSP50_5026)* | TGGCTCTGCGTGTGGACCT | CGGCGAAATTCGCATACCAG | 288 |
| *glgC* (*ACSP50_5028*) | GGATGATCGACTTCGTTCTC | GTGACGTAGTTGCCGAGCAG | 141 |
| *glgE (ACSP50_5262)* | GTGAACTGGTACCGGTTTCC | CGAACGCCTCGATCGTGAAC | 198 |
| *treS (ACSP50_5263)* | TGATCGAGCGCCTGGACTAC | GGACGTGTGGTTCATGAC | 218 |
| *mak/pep2* (*ACSP50_5264*) | CGTACACCGACGGCAGTTC | GGCTCCTTCTCGAAGGTCAG | 210 |
| *glgB (ACSP50_5265)* | ATGATCGGGCACACCACCTC | TCGATGTCGAGCCGGTAGTC | 263 |
| *pulA Z*(*ACSP50_6383*) | GGTGCTCAGCCTTCTTCTC | TTGCTCGCCGGCTTGTCG | 117 |
| *malG (ACSP50_6384)* | TGACCGCGAAATGGTTCAAG | CGGCGATCATCACCGAGTTG | 228 |
| *malF (ACSP50_6385)* | TGCGCAGCCAGAGCATCAAG | GATCGCCTCAGCCTTGTTGC | 290 |
| *malE (ACSP50_6386)* | ACCGGCGAGCTGATCATCTG | ACCAGGTTGCCGATCCAGTC | 203 |
| *acrC (ACSP50_ 6387)* | GAGCGACTGCTCAACAAG | GTCGTCGAAACCGATCAC | 132 |
| *treZ (ACSP50_6609)* | ATGACAACCTTCGAGGTATG | TGAAGGCGGCGTGGTCGTAG | 241 |
| *treY (ACSP50_6610)* | TCCAAGTCCGTCCCGACTT | CCGAGCCCGGCGTTCTTCAG | 220 |
| *treX* (*ACSP50_6611*) | CGACAACCGCACCTATTAC | CATCTCGGTAACCCAGTAG | 145 |
| *glgP (ACSP50_6911)* | TGCTGAGCAGCCTCTATTAC | AGTAGACCCACCGCGGATTG | 159 |
| *ppgK* (*ACSP50_7038*) | CAAGGGTTCGGTGCTGGATG | AGCTGCCCACCCAGATCC | 120 |
| *treS (ACSP50_7524)* | TGAGTGACCGGTGGTATCAG | TGAGTGACCGGTGGTATCAG | 294 |
| *malQ (ACSP50_7587)* | AGTCGGTGATCGGGGTGCTG | GGCCGGGACCACCACGAC | 245 |
| *otsB (ACSP50_7717)* | CGAGCAGCCGGAGGAGTC | GAGGCCGTAGAGGCCGAA | 166 |
| *gtaB/galU (ACSP50_7820)* | CTCGCCTTCATCGAGGTCAC | GGCGATCGTCTCGAAGATCC | 192 |
| *ACSP50_8228* | AGGACCCGACCGCGAACC | TTGGCCTGGTCGGCACAC | 232 |
| *ACSP50_8229* | GGCCGGCTCGCTATCGTG | AACTCGTCGAGCATGATCC | 239 |

**Supplementary Table 3.** Genes and their annotated function, genomic localization and operon organization, which are assumed to be involved in the maltose/maltodextrin and glycogen metabolism respectively carbophore metabolism of *Actinoplanes*sp. SE50/110. Homology comparisons to putative functional homologues from *C. glutamicum* and *E. coli* by BlastP (Altschul, S. et al. 1997; Altschul, Stephen et al. 2005) are documented by percentages of sequence identities and percentages of positives.

| **locus tag (*ACSP50*)** | **name** | **annotated function** | **position in the chromosome (start-stop) (GenBank: LT827010.1) (Wolf, T. et al. 2017)** | **Homology comparison by BlastP analyses (identities/positives) (Altschul, S. et al. 1997) to functional characterized orthologues from** | | | |
| --- | --- | --- | --- | --- | --- | --- | --- |
|  |  |  |  | ***C. glutamicum* ATCC 13032^TM^** | | ***E. coli (taxid:562)*** | |
|  | | | | | | | |
| **GlgE-and central metabolism** | | | | | | | |
| *1226* | ***otsA*** | trehalose-6-phosphate synthase | 1360350-1358953 | (53 %, 66 %) | (Wolf, A. et al. 2003; Tzvetkov et al. 2003) | (37 %, 53 %) | (Giaever et al. 1988; Kaasen et al. 1992) |
| *1604* | ***otsA*** | trehalose-6-phosphate synthase | 1780423-1779008 | (48 %, 62 %) |  | (39 %, 55 %) |  |
| *3343* | ***otsA*** | trehalose-6-phosphate synthase | 3750399-3748957 | (51 %, 63 %) |  | (38 %, 52 %) |  |
| *2104* | ***otsA*** | trehalose-6-phosphate synthase | 2337364-2338776 | (47 %, 60 %) |  | (35 %, 51 %) |  |
| *7717* | ***otsB*** | trehalose-phosphatase | 8510713-8511519 | no homology to corynebacterial otsB found |  | (30 %, 46 %) |  |
| *7524* | ***treS*** | trehalose synthase | 8313777-8312131 | (40 %, 57 %) | (Wolf, A. et al. 2003) | function not described for *E. coli* (41 %, 58 %) | |
| *5263* | ***treS*** | trehalose synthase | 5818367-5820070 | (70 %, 82 %) |  | not described for *E. coli* (37 %, 53 %) | |
| *5264* | ***pep2/mak*** | putative maltokinase | 5820067-5821380 | (54 %, 67 %) |  | function not described for *E. coli, no*  homologue found | |
| *6609* | ***treZ*** | malto-oligosyltrehalose trehalohydrolase | 7267181-7265448 | (50 %, 63 %) | (Tzvetkov et al. 2003) | not described for *E. coli* (39 %, 52 %) | |
| *6610* | ***treY*** | malto-oligosyltrehalose synthase | 7269445-7267178 | (44 %, 57 %) |  | not described for *E. coli* (37 %, 51 %) | |
| *5262* | ***glgE*** | alpha-amylase, alpha-1,4-glucan-maltose-1-phosphate maltosyltransferase | 5816338-5818302 | (54 %, 67 %) |  | no homology found | |
| *7820* | ***galU/gtaB*** | UTP-glucose-1-phosphate uridylyltransferase | 8628036-8627125 | (43 %, 61 %) |  | (44 %, 59 %) | (Hossain et al. 1994) |
| *2729* | ***pgmA*** | phosphoglucomutase | 3060148-3061767 | (69 %, 78 %) | (Seibold, Gerd M. und Eikmanns 2013) | (60 %, 73 %) | (Lu und Kleckner 1994) |
| *7747* | ***pgmA/pmmB*** | phosphomannomutase | 8547502-8549106 | (40 %, 52 %) |  | (27 %, 38 %) |  |
| *1600* | ***gck*** | glucose kinase | 1775218-1776165 | (41 %, 58 %) | (Park, S. et al. 2000) | (35 %, 47 %) | (Meyer et al. 1997) |
| *3554* |  | N-acetyl-D-glucosamine kinase | 4010429-4009875 | (32 %, 48 %) |  | (35 %, 49 %) |  |
| *7038* | ***ppgK*** | polyphosphate glucokinase | 5813745-5814596 | (50 %, 69 %) |  | (26 %, 42 %) |  |
| **GlgA-metabolism** | | | | | | | |
| *5026* | ***glgA*** | glycosyl transferase family 1, glycogen synthase | 5549384-5548197 | (53 %, 67 %) | (Tzvetkov et al. 2003) | (28 %, 44 %) | (Park, J.‑T. et al. 2011) |
| *5028* | ***glgC*** | glucose-1-phosphate adenylyltransferase | 5551726-5552958 | (64 %, 74 %) | (Seibold, Gerd et al. 2007) | (41 %, 62 %) | reviewed by (Boos, Winfried und Shuman, Howard 1998) |
| **degradation of branched α-glucans** | | | | | | | |
| *6611* | ***glgX/treX*** | glycogen-debranching enzyme | 7271556-7269445 | (64 %, 78 %) | (Seibold, Gerd M. und Eikmanns 2007) | (50 %, 65 %) | (Dauvillée et al. 2005) |
| *4618* | ***glgX*** | glycogen-debranching enzyme | 5103907-5101790 | (67 %, 80 %) |  | (54 %, 67 %) |  |
| *2754* | ***treX/glgX*** | glycogen-debranching enzyme | 3089503-3091611 | (64 %, 77 %) |  | (51 %, 64 %) |  |
| *6911* | ***glgP*** | glucan phosphorylase | 7628007-7630448 | (42 %, 59 %) | (Seibold, Gerd M. et al. 2009) | (47 %, 64 %) | (Alonso-Casajús et al. 2006; Park, J.‑T. et al. 2011) |
| *5265* | ***glgB*** | glycogen-branching enzyme | 5821407-5823557 | (54 %, 66 %) | (Seibold, Gerd M. et al. 2011) | (48 %, 62 %) | reviewed by (Boos, Winfried und Shuman, Howard 1998) |
| *5152* | ***glgB homologue?*** | hypothetical protein, 1,4-alpha-glucan-branching protein | 5694906-5692999 | (26 %, 38 %) |  | (25 %, 37 %) |  |

Supplementary Table 3 continued

| **maltose/maltodextrin metabolism** | | | | | | | |
| --- | --- | --- | --- | --- | --- | --- | --- |
| *4430* | ***malZ*** | alpha-amylase | 4906374-4907660 | not annotated (Seibold, Gerd M. et al. 2009) | | (28 %, 43 %) | (Song et al. 2010) |
| *2474* | ***amlE*** | alpha-glucosidase/maltase | 2756453-2754780 | not described for *C. glutamicum*, homology found to a glycosyl-transferase (29 %, 43 %) | | not described for *E. coli,* homology found to an alpha-phosphotrehalase  (36 %, 50 %) | |
| *1177* | genes with sequence homology to AmlE | alpha-glucosidase | 1313897-1312293 | see above (31 %, 45 %) | | see above (36 %, 50 %) | |
| *6814* |  | glucohydrolase | 7507856-7506156 | see above (28 %, 43 %) | | see above (45 %, 65 %) | |
| *6830* |  | alpha-glucosidase | 7528792-7527233 | see above (32 %, 47 %) | | see above (36 %, 50 %) | |
| *7587* | ***malQ*** | 4-alpha-glucanotransferase | 8377994-8379910 | (37 %, 52 %) | (Seibold, Gerd M. et al. 2009) | (33 %, 46 %) | (Park, J.‑T. et al. 2011) |
| *3601* | ***acbQ*** | acarbose 4-alpha-glucanotransferase, amlyomaltase | 4070451-4072547 | not described for *C. glutamicum,* homology *to* alpha-glucanotransferase (40 %, 55 %) | | not described for *E. colli, homology to* alpha-glucanotransferase (32 %, 48 %) | |
| **regulatory genes discussed in the context of sugar metabolism** | | | | | | | |
| *2475* | ***amlR*** | putative regulator of *aml* operon | 2757621-2756536 | not described for *C. glutamicum, homology to* LacI-type transcriptional regulator found (31 %, 47 %) | | not described for *E. coli, homology to* PurR transcriptional regulator found (33 %, 48 %) | |
| *6387* | ***acrC*** | transcriptional regulator; LacI family | 7038931-7039968 | not described for *C. glutamicum, homology to* lacI-type transcriptional regulator found (32 %, 46 %) | | (32 %, 52 %) |  |
| *3915* | ***malT*** | transcriptional regulator | 4404927-4407536 | not found | | (24 %, 41 %) | (Cole und Raibaud, Olivier 1986; Dardonville und Raibaud, O. 1990; Richet und Raibaud, O. 1987, 1989) |
| *3917* | ***malT homologue*** | MalT-like; LuxR family transcriptional regulator | 4408284-4410905 | not found | | (33 %, 46 %) |  |
| *4918* | ***malT homologue?*** | ATP-dependent transcriptional regulator; MalT-like; LuxR family | 5427736-5425160 | not found | | not found |  |
| **enzymes of carbophore model (Wehmeier, Udo 2003; Wehmeier, U. und Piepersberg 2004; Wendler et al. 2013).** | | | | | | | |
| *3590* | ***acbZ*** | acarbose-resistant alpha-amylase; pullulanase | 4054028-4057255 | not found | | not found | |
| *3610* | ***acbE*** | acarbose-resistant alpha-amylase; pullulanase | 4083616-4080515 | not found | | not found | |
| *6383* | ***pulA*** | pullulanase; alpha-amylase | 7035042-7029685 | not found | | not found | |
| *3611* | ***acbD*** | acarviose transferase | 4083942-4086116 | not found | | not found | |
| *3601* | ***acbQ*** | acarbose 4-alpha-glucanotransferase, amlyomaltase | 4070451-4072547 | not described for *C. glutamicum,* homology *to* alpha-glucanotransferase (40 %, 55 %) | | not described for *E. colli, homology to* alpha-glucanotransferase (32 %, 48 %) | |
| *3602* | ***acbK*** | acarbose 7-kinase | 4072579-4073478 | not found | | not found | |
| *3591-3* | ***acbWXY*** | ABC-type transporter, ATPase component and permease components | 4059993-4058920  4058923-4058120  4058123-4057320 | (33 %, 54 %)  not found  not found | | (41 %, 63 %)  (28 %, 46 %)  (39 %, 61 %) | |
| *3612-4* | ***galHFG*** | ABC-type galactose transporter, substrate-binding lipoprotein and permease components | 4089474-4088173  4088173-4087193  4087196-4086351 | not found  (31 %, 52 %)  (25 %, 48 %) | | (25 %, 44 %)  (32 %, 53 %)  (30 %, 53 %) | |
| *2505-7* | ***aglEFG*** | ABC-type multiple alpha-glucoside transporter, substrate-binding lipoprotein and permease components | 2795669-2794296  2794199-2793204  2793204-2792293 | not found  (31 %, 54 %)  (34 %, 52 %) | | not found  (30 %, 52 %)  (33 %, 59 %) | |
| *6384-6* | ***malEFG*** | ABC-type maltose/maltodextrin transporter, substrate-binding lipoprotein and permease components | 7038735-7037482  7037482-7035926  7035929-7035078 | not found  (24 %, 42 %)  (31 %, 56 %) | | (32 %, 47 %)  (35 %, 55 %)  (37 %, 61 %) | |

1.3 References

Alonso-Casajús, Nora; Dauvillée, David; Viale, Alejandro Miguel; Muñoz, Francisco José; Baroja-Fernández, Edurne; Morán-Zorzano, María Teresa et al. (2006): Glycogen phosphorylase, the product of the glgP Gene, catalyzes glycogen breakdown by removing glucose units from the nonreducing ends in Escherichia coli. In: *Journal of bacteriology* 188 (14), S. 5266–5272. DOI: 10.1128/JB.01566-05.

Altschul, S. F.; Madden, T. L.; Schäffer, A. A.; Zhang, J.; Zhang, Z.; Miller, W.; Lipman, D. J. (1997): Gapped BLAST and PSI-BLAST: a new generation of protein database search programs. In: *Nucleic acids research* 25 (17), S. 3389–3402.

Altschul, Stephen F.; Wootton, John C.; Gertz, E. Michael; Agarwala, Richa; Morgulis, Aleksandr; Schäffer, Alejandro A.; Yu, Yi-Kuo (2005): Protein Database Searches Using Compositionally Adjusted Substitution Matrices. In: *The FEBS journal* 272 (20), S. 5101–5109. DOI: 10.1111/j.1742-4658.2005.04945.x.

Boos, Winfried; Shuman, Howard (1998): Maltose/Maltodextrin System of Escherichia coli: Transport, Metabolism, and Regulation. In: *Microbiology and molecular biology reviews : MMBR* 62 (1), S. 204–229.

Chandra, Govind; Chater, Keith F.; Bornemann, Stephen (2011): Unexpected and widespread connections between bacterial glycogen and trehalose metabolism. In: *Microbiology (Reading, England)* 157 (Pt 6), S. 1565–1572. DOI: 10.1099/mic.0.044263-0.

Cole, Stewart T.; Raibaud, Olivier (1986): The nucleotide sequence of the malT gene encoding the positive regulator of the Escherichia coli maltose regulon. In: *Gene* 42 (2), S. 201–208. DOI: 10.1016/0378-1119(86)90297-0.

Dardonville, B.; Raibaud, O. (1990): Characterization of malT mutants that constitutively activate the maltose regulon of Escherichia coli. In: *Journal of bacteriology* 172 (4), S. 1846–1852.

Dauvillée, David; Kinderf, Isabelle S.; Li, Zhongyi; Kosar-Hashemi, Behjat; Samuel, Michael S.; Rampling, Lynette et al. (2005): Role of the Escherichia coli glgX Gene in Glycogen Metabolism. In: *Journal of bacteriology* 187 (4), S. 1465–1473. DOI: 10.1128/JB.187.4.1465-1473.2005.

Giaever, H. M.; Styrvold, O. B.; Kaasen, I.; Strøm, A. R. (1988): Biochemical and genetic characterization of osmoregulatory trehalose synthesis in Escherichia coli. In: *Journal of bacteriology* 170 (6), S. 2841–2849. DOI: 10.1128/jb.170.6.2841-2849.1988.

Hossain, S. A.; Tanizawa, K.; Kazuta, Y.; Fukui, T. (1994): Overproduction and characterization of recombinant UDP-glucose pyrophosphorylase from Escherichia coli K-12. In: *Journal of biochemistry* 115 (5), S. 965–972.

Kaasen, I.; Falkenberg, P.; Styrvold, O. B.; Strøm, A. R. (1992): Molecular cloning and physical mapping of the otsBA genes, which encode the osmoregulatory trehalose pathway of Escherichia coli: evidence that transcription is activated by katF (AppR). In: *Journal of bacteriology* 174 (3), S. 889–898.

Kalscheuer, Rainer; Syson, Karl; Veeraraghavan, Usha; Weinrick, Brian; Biermann, Karolin E.; Liu, Zhen et al. (2010): Self-poisoning of Mycobacterium tuberculosis by targeting GlgE in an alpha-glucan pathway. In: *Nature chemical biology* 6 (5), S. 376–384. DOI: 10.1038/nchembio.340.

Koliwer-Brandl, Hendrik; Syson, Karl; van de Weerd, Robert; Chandra, Govind; Appelmelk, Ben; Alber, Marina et al. (2016): Metabolic Network for the Biosynthesis of Intra- and Extracellular α-Glucans Required for Virulence of Mycobacterium tuberculosis. In: *PLoS pathogens* 12 (8), e1005768. DOI: 10.1371/journal.ppat.1005768.

Lee, Jin-Sook; Hai, Tran; Pape, Hermann; Kim, Tae-Jong; Suh, Joo-Won (2008): Three trehalose synthetic pathways in the acarbose-producing Actinoplanes sp. SN223/29 and evidence for the TreY role in biosynthesis of component C. In: *Applied microbiology and biotechnology* 80 (5), S. 767–778. DOI: 10.1007/s00253-008-1582-3.

Love, Michael; Anders, Simon; Huber, Wolfgang (2017): DESeq2: Bioconductor.

Lu, M.; Kleckner, N. (1994): Molecular cloning and characterization of the pgm gene encoding phosphoglucomutase of Escherichia coli. In: *Journal of bacteriology* 176 (18), S. 5847–5851.

Meyer, D.; Schneider-Fresenius, C.; Horlacher, R.; Peist, R.; Boos, W. (1997): Molecular characterization of glucokinase from Escherichia coli K-12. In: *Journal of bacteriology* 179 (4), S. 1298–1306.

Miah, Farzana; Bibb, Maureen J.; Barclay, J. Elaine; Findlay, Kim C.; Bornemann, Stephen (2016): Developmental delay in a Streptomyces venezuelae glgE null mutant is associated with the accumulation of α-maltose 1-phosphate. In: *Microbiology (Reading, England)* 162 (7), S. 1208–1219. DOI: 10.1099/mic.0.000296.

Park, Jong-Tae; Shim, Jae-Hoon; Tran, Phuong Lan; Hong, In-Hee; Yong, Hwan-Ung; Oktavina, Ershita Fitria et al. (2011): Role of maltose enzymes in glycogen synthesis by Escherichia coli. In: *Journal of bacteriology* 193 (10), S. 2517–2526. DOI: 10.1128/JB.01238-10.

Park, S. Y.; Kim, H. K.; Yoo, S. K.; Oh, T. K.; Lee, J. K. (2000): Characterization of glk, a gene coding for glucose kinase of Corynebacterium glutamicum. In: *FEMS Microbiology Letters* 188 (2), S. 209–215. DOI: 10.1111/j.1574-6968.2000.tb09195.x.

Richet, E.; Raibaud, O. (1987): Purification and properties of the MalT protein, the transcription activator of the Escherichia coli maltose regulon. In: *Journal of Biological Chemistry* 262 (26), S. 12647–12653.

Richet, E.; Raibaud, O. (1989): MalT, the regulatory protein of the Escherichia coli maltose system, is an ATP-dependent transcriptional activator. In: *The EMBO Journal* 8 (3), S. 981–987.

Schneider, D.; Bruton, C. J.; Chater, K. F. (2000): Duplicated gene clusters suggest an interplay of glycogen and trehalose metabolism during sequential stages of aerial mycelium development in Streptomyces coelicolor A3(2). In: *Molecular & general genetics : MGG* 263 (3), S. 543–553.

Seibold, Gerd; Dempf, Stefan; Schreiner, Joy; Eikmanns, Bernhard J. (2007): Glycogen formation in Corynebacterium glutamicum and role of ADP-glucose pyrophosphorylase. In: *Microbiology (Reading, England)* 153 (Pt 4), S. 1275–1285. DOI: 10.1099/mic.0.2006/003368-0.

Seibold, Gerd M.; Breitinger, Katrin J.; Kempkes, Raoul; Both, Leonard; Krämer, Matthias; Dempf, Stefan; Eikmanns, Bernhard J. (2011): The glgB-encoded glycogen branching enzyme is essential for glycogen accumulation in Corynebacterium glutamicum. In: *Microbiology (Reading, England)* 157 (Pt 11), S. 3243–3251. DOI: 10.1099/mic.0.051565-0.

Seibold, Gerd M.; Eikmanns, Bernhard J. (2007): The glgX gene product of Corynebacterium glutamicum is required for glycogen degradation and for fast adaptation to hyperosmotic stress. In: *Microbiology (Reading, England)* 153 (Pt 7), S. 2212–2220. DOI: 10.1099/mic.0.2006/005181-0.

Seibold, Gerd M.; Eikmanns, Bernhard J. (2013): Inactivation of the phosphoglucomutase gene pgm in Corynebacterium glutamicum affects cell shape and glycogen metabolism. In: *Bioscience reports* 33 (4). DOI: 10.1042/BSR20130076.

Seibold, Gerd M.; Wurst, Martin; Eikmanns, Bernhard J. (2009): Roles of maltodextrin and glycogen phosphorylases in maltose utilization and glycogen metabolism in Corynebacterium glutamicum. In: *Microbiology (Reading, England)* 155 (Pt 2), S. 347–358. DOI: 10.1099/mic.0.023614-0.

Song, Kyung-Mo; Shim, Jae-Hoon; Park, Jong-Tae; Kim, Sung-Hee; Kim, Young-Wan; Boos, Winfried; Park, Kwan-Hwa (2010): Transglycosylation properties of maltodextrin glucosidase (MalZ) from Escherichia coli and its application for synthesis of a nigerose-containing oligosaccharide. In: *Biochemical and biophysical research communications* 397 (1), S. 87–92. DOI: 10.1016/j.bbrc.2010.05.073.

Tapio, S.; Yeh, F.; Shuman, H. A.; Boos, W. (1991): The malZ gene of Escherichia coli, a member of the maltose regulon, encodes a maltodextrin glucosidase. In: *Journal of Biological Chemistry* 266 (29), S. 19450–19458.

Tzvetkov, Mladen; Klopprogge, Corinna; Zelder, Oskar; Liebl, Wolfgang (2003): Genetic dissection of trehalose biosynthesis in Corynebacterium glutamicum: inactivation of trehalose production leads to impaired growth and an altered cell wall lipid composition. In: *Microbiology (Reading, England)* 149 (Pt 7), S. 1659–1673. DOI: 10.1099/mic.0.26205-0.

Wehmeier, U. F.; Piepersberg, W. (2004): Biotechnology and molecular biology of the alpha-glucosidase inhibitor acarbose. In: *Applied microbiology and biotechnology* 63 (6), S. 613–625. DOI: 10.1007/s00253-003-1477-2.

Wehmeier, Udo F. (2003): The Biosynthesis and Metabolism of Acarbose in Actinoplanes sp. SE 50/110: A Progress Report. In: *Biocatalysis and Biotransformation* 21 (4-5), S. 279–284. DOI: 10.1080/10242420310001614388.

Wendler, Sergej; Hürtgen, Daniel; Kalinowski, Jörn; Klein, Andreas; Niehaus, Karsten; Schulte, Fabian et al. (2013): The cytosolic and extracellular proteomes of Actinoplanes sp. SE50/110 led to the identification of gene products involved in acarbose metabolism. In: *Journal of biotechnology* 167 (2), S. 178–189. DOI: 10.1016/j.jbiotec.2012.08.011.

Wilson, Wayne A.; Roach, Peter J.; Montero, Manuel; Baroja-Fernández, Edurne; Muñoz, Francisco José; Eydallin, Gustavo et al. (2010): Regulation of glycogen metabolism in yeast and bacteria. In: *FEMS microbiology reviews* 34 (6), S. 952–985. DOI: 10.1111/j.1574-6976.2010.00220.x.

Wolf, Andreas; Krämer, Reinhard; Morbach, Susanne (2003): Three pathways for trehalose metabolism in Corynebacterium glutamicum ATCC13032 and their significance in response to osmotic stress. In: *Molecular microbiology* 49 (4), S. 1119–1134. DOI: 10.1046/j.1365-2958.2003.03625.x.

Wolf, Timo; Schneiker-Bekel, Susanne; Neshat, Armin; Ortseifen, Vera; Wibberg, Daniel; Zemke, Till et al. (2017): Genome improvement of the acarbose producer Actinoplanes sp. SE50/110 and annotation refinement based on RNA-seq analysis. In: *Journal of biotechnology*. DOI: 10.1016/j.jbiotec.2017.04.013.

Woo, Han Min; Noack, Stephan; Seibold, Gerd M.; Willbold, Sabine; Eikmanns, Bernhard J.; Bott, Michael (2010): Link between phosphate starvation and glycogen metabolism in Corynebacterium glutamicum, revealed by metabolomics. In: *Applied and environmental microbiology* 76 (20), S. 6910–6919. DOI: 10.1128/AEM.01375-10.
